# Back(s) to basics: The concept of backing in stone tool technologies for tracing hominins' technical innovations

**DOI:** 10.1002/evan.22045

**Published:** 2024-08-07

**Authors:** Davide Delpiano, Brad Gravina, Marco Peresani

**Affiliations:** ^1^ Dipartimento di Studi Umanistici, Sezione di Scienze Preistoriche e Antropologiche Università degli Studi di Ferrara Ferrara Italy; ^2^ Musée National de Préhistoire Les Eyzies France; ^3^ PACEA, UMR 5199, CNRS, Université de Bordeaux, Bât B2, Allée Geoffroy Saint‐Hilaire CS 50023 Pessac France; ^4^ Istituto di Ingegneria Ambientale e Geoingegneria, Consiglio Nazionale delle Ricerche Milano Italy

**Keywords:** discoidal, ergonomics, hafting, Levallois, Paleolithic, technical innovations, tool backing

## Abstract

The evolution of Paleolithic stone tool technologies is characterized by gradual increase in technical complexity along with changes in the composition of assemblages. In this respect, the emergence of retouched‐backed tools is an important step and, for some, a proxy for “modern” behavior. However, backed tools emerge relatively early and develop together with major changes in Middle‐Upper Pleistocene stone tool technologies. We provide an updated review of the emergence and development of the “backing” concept across multiple chrono‐cultural contexts and discuss its relationship to both the emergence of hafting and major evolutionary steps in the ergonomics of stone tool use. Finally, we address potential mechanisms of context‐specific re‐invention of backing based primarily on data from the late Middle Paleolithic of Western Europe.

## 1 INTRODUCTION

The nature and form of stone tools (i.e., type fossils) as well as associated production methods are commonly used to define Paleolithic techno‐complexes, trace cultural shifts, and track the emergence of innovations in Prehistory. The evolution of knapping methods and techniques are generally characterized by a gradual increase in technical complexity, as reflected in the length and hierarchization of production sequences, in addition to how well they respond to production objectives in terms of shape, functionality, and efficiency (e.g., Refs.^1^, ^2^, ^3^, ^4^, ^5^).

The ways in which stone tools were conceived, produced, and used also likely reflects cultural traditions, becoming increasingly codified and standardized with the emergence of the Upper Paleolithic. These traditions can be addressed in terms of knowledge or information transmission as well as ecological perspectives that incorporate a consideration of resource affordances and the constraints of the specific environmental setting of tool production and use. As such, shifts in knapping processes and the composition of stone tool assemblages are often considered archeological proxies for tracing both cognitive and biological shifts as well as technological change driven by the emergence of new populations, demographic fluctuations, migrations, or ecological and environmental factors.

However, identifying the precise steps or trajectories of technical development and innovation in early Prehistory is difficult. In the case of traditional societies, which tend to be more conservative, traditions of tool manufacture are transmitted across generations, gradually become stable and accepted cultural elements.^6^ This scenario of long‐lasting stability (e.g., Lower Paleolithic bifacial tools) does not, however, exclude innovation and invention. Technological development throughout history, as well as in prehistoric societies, can unfold following two main processes^7^: (1) by saturation of a technical scheme of production, which is stabilized through the universal convergence of types having the same techno‐functional aim, leading to a standardization of the product; or (2) by remodeling the scheme after the emergence of an invention (e.g., the creation of a new tool with different functions) or an innovation. In other words, the use of a new method of production/configuration to transform an existing technical element into a more effective version.

Cultural transmission in these societies involves the transfer of information, skills, knowledge, or behaviors via three main channels: vertical transmission (from parents to children), horizontal transmission (between members of the same generation), oblique transmission (between members of different generations, not involving parents).^8^ Experimental studies have reinforced the role of complex teaching‐learning mechanisms, that is, active teaching, possibly involving verbal communication.^9^, ^10^ In fact, for lithic technologies, imitation, and emulation alone do not suffice for the successful transfer of knowledge and innovations.^11^ In human societies, high‐fidelity transmission underlies the development of cumulative culture, which is enhanced by each generation if the adequate cognitive, demographic, and social conditions are in place.^12^ High‐fidelity transmission equally ensures that innovations are passed on and eventually stabilized. However, in the absence of a coding method facilitating this process, technical innovations are less susceptible to being adopted and transmitted and conversely forgotten.

Notwithstanding clear problems with the term, the technological transition towards “modernity” is an instructive example. Several models of behavioral change highlight the sudden or gradual replacement of diagnostic tool types as a key feature of cultural changeover.^4^, ^13^, ^14^, ^15^, ^16^ However, growing archeological data point to a more mosaic and patchy scenario of cultural change, implying periods of stasis or even the disappearance of innovative traits rather than incremental developments.^17^, ^18^, ^19^ Innovations in prehistoric societies, for example, adhesives, can emerge in a punctuated or episodic manner, especially in the absence of developed networks of communication connecting human groups or means to retain information. For an innovation to be assimilated, it needs to find fertile ground in the society where it is developed.^20^, ^21^ Technical objects or processes are designed to respond to human needs in the most efficient means possible^20^; however, the “need” is not necessarily a fixed quantity, and can change substantially according to how societies are organized. This would explain why in certain societies, particular innovations fail to take hold because their enhanced efficiency is un‐ or under‐exploited. Demographic and social factors also play a role: technological shifts are fostered and accelerated by the connectivity and mobility of hunter‐gatherer groups.^8^, ^22^, ^23^, ^24^ While larger population sizes and fuller network interconnectivity reduce the risk of cultural loss or drift, they also significantly reduce diversity by homogenizing traits due to sustained group‐wide transmission.^25^, ^26^

This brings us to a second scenario commonly referred to as the convergence model. In this case, independent and context‐specific re‐inventions appear in differentiated metapopulations potentially favored by concomitant effectiveness of information transmission in analogous social and environmental contexts. However, archeologically discontinuous phenomena may also reflect research biases or be shaped by latent and independent behavioral solutions. Tracing a particular innovation over time and space can therefore help shed light on how and under what circumstances human groups developed consistent solutions to everyday problems in early Prehistory, providing a new interpretative key to better situate important steps of hominin behavioral evolution.

Due to their abundance and preferential preservation, stone tools are the primary evidence for processes of cultural and techno‐behavioral change in Lower and Middle Paleolithic. Mapping changes in a specific tool type or form over time within the larger context of technological change across the Lower to the Upper Pleistocene can provide a technologically informed perspective on changing biological, social, and behavioral dynamics. Here we address this issue by focusing on a particular Paleolithic tool form and edge modification—edge backing and backed tools, technical traits that have been linked to modern cognitive abilities.^15^, ^17^ Viewed from an interdisciplinary perspective, the spatiotemporal patterning in the appearance of these tools can provide a new dimension in the debate concerning the emergence of innovative cultural elements.

## 2 THE CONCEPT OF “BACKING” STONE TOOLS AND METHODOLOGICAL APPROACHES: A CASE STUDY

**Figure 1:**
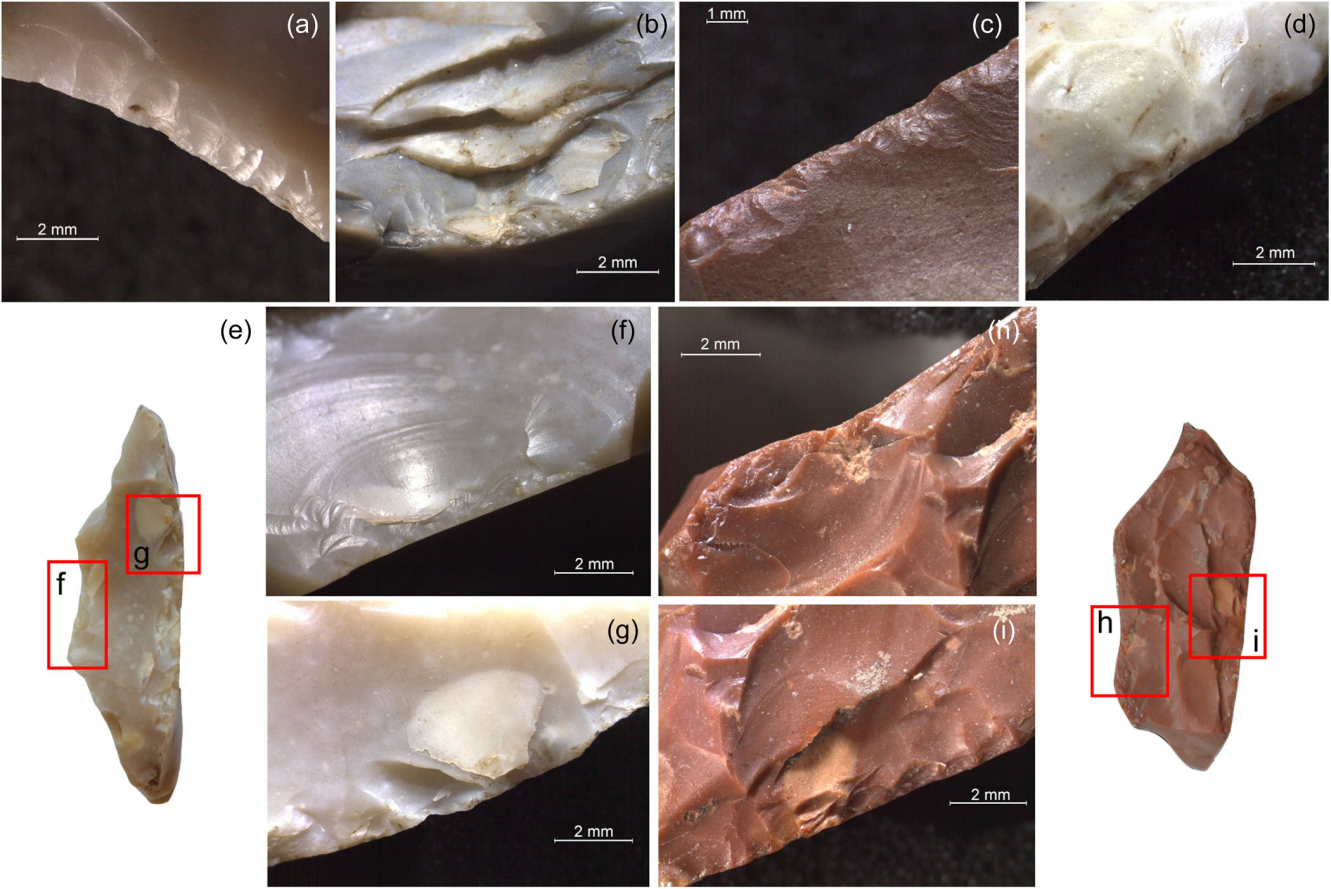
Modified backs from Fumane, layer A9: the morpho‐technical features suggest diverse retouch techniques, namely percussion/pressure with soft stone (a), percussion with hard hammer (b), abrasion (c), percussion followed by abrasion (d) and bipolar percussion on an anvil (e–h). *Source*: Modified after Delpiano^27^.

Although backed tools are relatively ubiquitous throughout the Paleolithic, there is little consensus in the literature concerning their precise definition. According to a techno‐functional perspective, a backed tool would be any asymmetric lithic artifact with a blunt, abrupt edge approaching 90° (i.e., the back) opposite a cutting/working edge. This definition is independent from the origin of the back, which can be natural (a cortical or natural surface), predetermined at detachment (i.e., a “relic core‐edge”), or retouched using a variety of techniques (Figure 1). As such, this single “type” subsumes a large spectrum of artifacts whose nature, form, and function likely differ substantially, ranging from heavy‐duty tools (e.g., flake cleavers) to light projectiles (e.g., micro‐Gravettes).

In our literature review, we paid particular attention to the appearance of the backing concept, defined here as the intentional modification or retouching of the back for ergonomic and/or functional ends. These “backed” artifacts fall under multiple terms that vary according to the chrono‐cultural context, region, and outline/shape, as well as different research traditions (Table 1). For example, François Bordes,^28^ in his typology of European Lower and Middle Paleolithic stone tools, distinguished typical and atypical backed knives based on the presence, extension, and regularity of retouch: naturally backed knives, backed and obliquely truncated points, backed scrapers, backed blades, and backed bifaces (*Keilmesser*s, *Prodniks*, etc.), sometimes singling out particular forms, for example, Acheulean backed knives or Audi knives, terms that are rarely used today (Table 1). Some authors distinguish “dos abattu” (abruptly retouched back) from a “dos retouché” (retouched back) according to the backed segment's positioning with respect to the other parts of the tool, with the term “dos façonné” (shaped back) including both variants.^39^

**Table 1:** Lower and Middle Paleolithic backed tools: Techno‐typology and terminology.

Technical terminology		
Abrupt/Semi‐abrupt retouch	Terms referring to the angle of retouch or removal. Abrupt is approximately 90° while semi‐abrupt is approximately 45°. Abrupt retouch shapes a back or a backed edge.	Inizan et al.^29^
Back	A general morphological term describing a surface that extends along the length of a blank that is more or less perpendicular to the two faces. This surface can be cortical, unretouched, prepared, or formed by abrupt retouch.	Inizan et al.^29^
Backed (edge)	An edge is considered to be backed when the continuous regular retouch applied to it is abrupt enough to rule out it being the active part of the tool (i.e., a cutting edge). An edge can be backed by abrupt or semi‐abrupt retouch modifying an unretouched edge, a cortical edge, and so on.	Inizan et al.^29^
Backed flake	Flake with an asymmetric cross‐section, a maximum thickness opposite the cutting edge corresponding to the back (natural or flaked surface) or the striking platform.	Turq^30^
Backed tool	Flakes or blades with steep retouch along one or more lateral edge.	De‐Sonneville Bordes & Perrot^31^
Backing/blunting	The action of shaping a backed edge by removing a series of small flakes along an edge with right angle (abrupt or semi‐abrupt retouch), or by breaking it against a hard surface (e.g., on an anvil). The edge is blunted to make it safer and easier to hold, apply pressure or to insert in a haft.	Barham^32^
Truncation	A line of regular continuous retouch, almost always abrupt, truncating either the proximal, distal, or lateral part of a flake, blade, or bladelet, forming two angles with the edges of the blank to which it is applied.	Inizan et al.^29^
Techno‐typology		
Flakes and blades with abrupt retouch (*éclats et lames a retouche abrupte ou alterne épaisse*)	Often pseudo‐tools. Blanks with “thick,” frequently alternate retouch.	Bordes^33^
Truncated flakes and blades (*éclats et lames tronqués*)	Truncation through abrupt or semi‐abrupt retouch on a distal end. This can be aligned or oblique to the axis of the blank, and straight, concave, or convex in profile.	Bordes^33^
Typical backed knife (*couteau à dos typique*)	Tool on a flake or blade with an unmodified cutting edge opposite an edge completely modified by continuous abrupt retouching (the back—*dos*).	Bordes^33^
Acheulean subtype	Large but not very abrupt back. Generally short and thick, often terminating ends in a preudo‐endscraper.	
Abri Audi subtype	On a large, thinner flake, with a more abrupt and curved back.	
Evolved subtype	On elongated flake/blade with very thick back tending to the resemble a Chatelperronian knife/point.	
Chatelperron knife	On small pointed blade, with thick and curved back.	
Atypical backed knife (*couteau à dos atypique*)	Tool on a flake or blade with an unmodified cutting edge opposite to an edge partially modified by abrupt or semi‐abrupt retouch.	Bordes^33^
Natural backed knife (*couteau à dos naturel*)	Tool on flake or blade with an unretouched cutting edge opposed to a cortical blunt edge acting as the back due to its angle (~90°) in respect to the lower surface.	Bordes^33^; Turq^30^
*Couteau à dos naturel enveloppant*	Characterized by a cortical striking platform extending along the entire or about half of the flake periphery.	
Core‐edge flake (*éclat débordant*)	Flake which retains a significant portion of the core's edge as a lateral extension of the platform. This lateral core‐edge bears either a bulb negative or flaked surface Also called a core trimming element.	Debenath & Dibble^34^; Turq^30^.
Pseudo‐Levallois point	Particular subtype of the debordant flake exhibiting a subtriangular shape with a lateral point shaped by convergent, unipolar removals opposite a back formed by a core edge, usually a bulb negative.	Debenath & Dibble^34^; Peresani^35^
Backed biface/*Keilmesser*	Asymmetric, bifacially shaped tools with a single cutting edge formed by bifacial flat‐convex retouch, opposite an unworked or minimally worked back.	Bordes^33^; Joris^36^
Microliths	Small flake‐ and blade‐based stone tools with bipolar blunting along one margin	Perera et al.^37^
Microlithic technology	Techno‐typological package including different components: (1) systematic production of small flakes, (2) use of backing (abrupt) retouch, including the production of geometric forms, (3) bladelet production from prismatic cores.	Pargeter^38^
Geometric piece	Generally, small tool made on fragments of small blades or bladelet. Abrupt retouch delimits a geometric form while one of the sides generally remains unretouched	De‐Sonneville Bordes & Perrot^31^
Segment/lunate/crescent	Geometric piece with a convex backed edge running along its full length	De‐Sonneville Bordes & Perrot^31^

In the “transition” between the Middle and Upper Paleolithic in Eurasia and especially between Middle Stone Age (MSA) and Later Stone Age (LSA) in Africa, the term “microlith” is sometimes ambiguously used as a synonym for backed tools, which are only a specific sub‐class of microlithic assemblages (Table 1). This term, primarily used for Late MSA, Early LSA, and Early Upper Paleolithic assemblages of Africa and South Asia, generally characterizes assemblages that may or may not include backed tools, which are often but not systematically made on microlithic blanks (see Pargeter^38^ for extensive bibliographic review). According to Perera et al.^37^ “microliths […are…] small flake‐ and blade‐based stone tools with bipolar blunting along one margin,” therefore including, by definition, a retouched back. However, following the criteria proposed by Pargeter and Shea,^40^ the term is equally employed to indicate the production of small blanks and is therefore not limited to uniquely describing backed pieces.

Terminological issues aside, every stone tool, retouched or unmodified, includes a passive or prehensile part that either is gripped in the hand or is in contact with a haft. The size and shape of the passive/prehensile portion is of key significance in terms of ergonomics, conditioning the functionality and the effectiveness of the tool, regardless of handedness. The backing of lithic artifacts has also been considered a marker of behavioral modernity and a proxy for cultural complexity^15^ given that (1) it is widely adopted and its use is consolidated within cultural techno‐complexes associated with anatomically modern humans in the African Middle Stone Age and Eurasian Early Upper Paleolithic and (2) it is commonly associated with the hafting of stone tools that may represent projectile elements. This has led some to limit this association of “modernity” and backed tools to the hafting of points or barbs in mechanically delivered weapons.^41^

The configuration and accommodation of the prehensile portion of stone tools is, however, present in earlier contexts, developing in different forms and proportions throughout the Lower and the Middle Paleolithic. This innovative feature is directly related to other physical, ergonomic, and economic aspects, such as more easily affording an efficient precision grip, facilitating the most effective transfer of force, and reducing time investment in the case of haft production and maintenance as well as retooling. These considerations are in turn tied to several characteristics of tool production, such as miniaturization, or technical innovation (e.g., tool hafting).^40^, ^42^, ^43^, ^44^

This being the case, it is important to bear in mind that edge angle and thickness alone are not sufficient to identify the passive (prehensile or hafted) area of a tool. For example, relatively abrupt “scraper retouch” can be confused for “backing”; in this case, the general shape of the tool and the interrelationship between the tool's areas or subsystems can help infer potential functions.^39^ For this reason, a systematic techno‐functional approach involving the de‐structuration of a tool's different elements into different (but also changeable) functional and interrelated relationships can be instructive.^45^, ^46^ This approach can connect the technological, morphometrical, and functional aspects of the tool, hypothetically decoding their potentialities, purposes, and uses. Of course, this approach reaches its fullest potential when complemented by technological and use‐wear analyzes and placed against the backdrop of the overall social‐techno‐cultural system.

Attribute analyzes of backed knives from different techno‐complexes exist, although they remain limited to a few regions and periods. These include Ruebens et al.^47^ investigation of the persistence of backed tools across the Middle‐to‐Upper Paleolithic transition of Western Europe to explore possible connections between the Châtelperronian and the local Mousterian of southwestern France, an analysis of backed tools to trace Late Stone Age cultural expressions in Africa,^48^ and the Middle to Late Stone Age transition in the Horn of Africa.^49^ Here, we present a critical review of backed tools in the broadest sense of the term, placing particular emphasis on the morpho‐technical and conceptual shifts in the gradual increase in their effectiveness or specific technical innovations. To do this, we created a database of sites where retouched backed tools have been reported, considering primarily qualitative data concerning the technological and chrono‐cultural context, the morphology and technology of the blanks selected for backing, the type, extension, position of the retouched back and, when available, inferences concerning tool function from use‐wear, residue analysis, and techno‐functional approaches (Supporting Information S1: File S1). To better frame variability in “backing” among late Neanderthals, we compared two late Middle Paleolithic assemblages from western Europe where these tools are particularly well attested: Fumane layer A9 and La Rochette layer 7^27^, ^50^ (see Supporting Information S1: File S2 for detailed data on the sites). The comparison was developed on different scales: techno‐functional analysis (sensu Lepot and Boëda^45^, ^46^ was applied to integrate technological, morphological, and functional aspects. Several morpho‐technical attributes were recorded on the tools, as well as their backs and cutting edges. The analysis allowed us to develop recurrent techno‐functional schemes. To conduct data management, analysis, charting, and basic statistics, we primarily utilized the R environment, specifically leveraging various packages such as “ggplot2,” “MASS,” and “scales” within RStudio Version 1.4.1106. Our overall goal is to reconstruct the dynamics underlying the appearance and spread of retouched backed tools, exploring their relationships with changes in the overall mechanics of tools prehension, ergonomics (i.e., from power grips to precision grasp to hafting arrangements), and miniaturization, possibly linking these techno‐behavioral shifts with Pleistocene biological and cognitive developments.

## 3 THE EMERGENCE OF THE BACKING “CONCEPT” IN THE EARLY AND MIDDLE PLEISTOCENE: NATURAL OR UNMODIFIED PREHENSILE AREAS

The creation or identification of a prehensile area in the earliest stone tools was probably conditioned by hand‐tool ergonomics and the morphological relationship with the cutting or working edge(s). The preferential selection of artifacts with a cortical or “angular” portion opposite a cutting edge appears to be already present with the early Oldowan ~2.3 Ma,^51^ suggesting that the utility of a prehensile “nonworking edge” was understood by hominin groups during the earliest phases of human evolution.

These early “naturally backed” implements are gradually replaced by tools that reflect a more concrete expression of the backing “concept” with the emergence of the Acheulean. This technical, and perhaps cognitive shift, potentially reflects increased “intentionality” in the conception and development of stone tool production methods already from 1.4 to 1.3 Ma.^52^ In the case of backed artifacts, two possible procedures can be used^53^; the production of wedged‐shaped blanks involving limited shaping or lateral detachments from simple single‐surface cores following a more elaborate knapping sequence.

Elements of tool standardization appear relatively early in the Lower Paleolithic, including the differentiation between a back (or prehensile portion) and an active cutting edge. Large cutting tools (bifaces and flake cleavers) are sometimes entirely shaped, with the prehensile part often intentionally thinned or modified by retouch. Key^54^ found that cutting efficiency was greater in bifaces with large edge angles in their basal portions, which they connected to the tools being more easily manipulated thus allowing more force to applied with the tip. Moreover, compared to other tools forms, bifaces permit substantial diversity in terms of grip, which has been experimentally demonstrated to vary in the same individuals and during the same task.^55^ This diversity may be related to the fact that bifaces sometimes lack a clear, ergonomic prehensile portion.

On the other hand, backed knives are common among Acheulean large cutting tools. Usually made on large flakes and with pointed or crescent‐like morphologies, these tools are characterized by asymmetrical cross‐sections defining a backed edge, which could originate either from cortical surfaces, fracture planes in raw materials, striking platforms, or intentional shaping.^52^, ^56^ A recent study of a late Acheulean assemblage from Wonderboom, South Africa, found that backed knives were made without any specific shaping but concerned diverse blanks and the opportunistic recycling of waste products. Any further shaping of blanks was only noted among pointed tools. This pattern, possibly associated with an early Middle Pleistocene member of Homo suggests the recognition of suitable an active edge‐prehensile area configuration of unmodified debitage products.^53^ In Western European Lower Paleolithic assemblages with or without bifaces (MIS 14‐11), the blanks selected for retouching often had a natural back (see Connet et al.^57^ for review). The distribution of the center of mass in these tools was weighted toward the backed edge, which likely increased manual control over cutting and/or scraping actions.^54^

**Figure 2:**
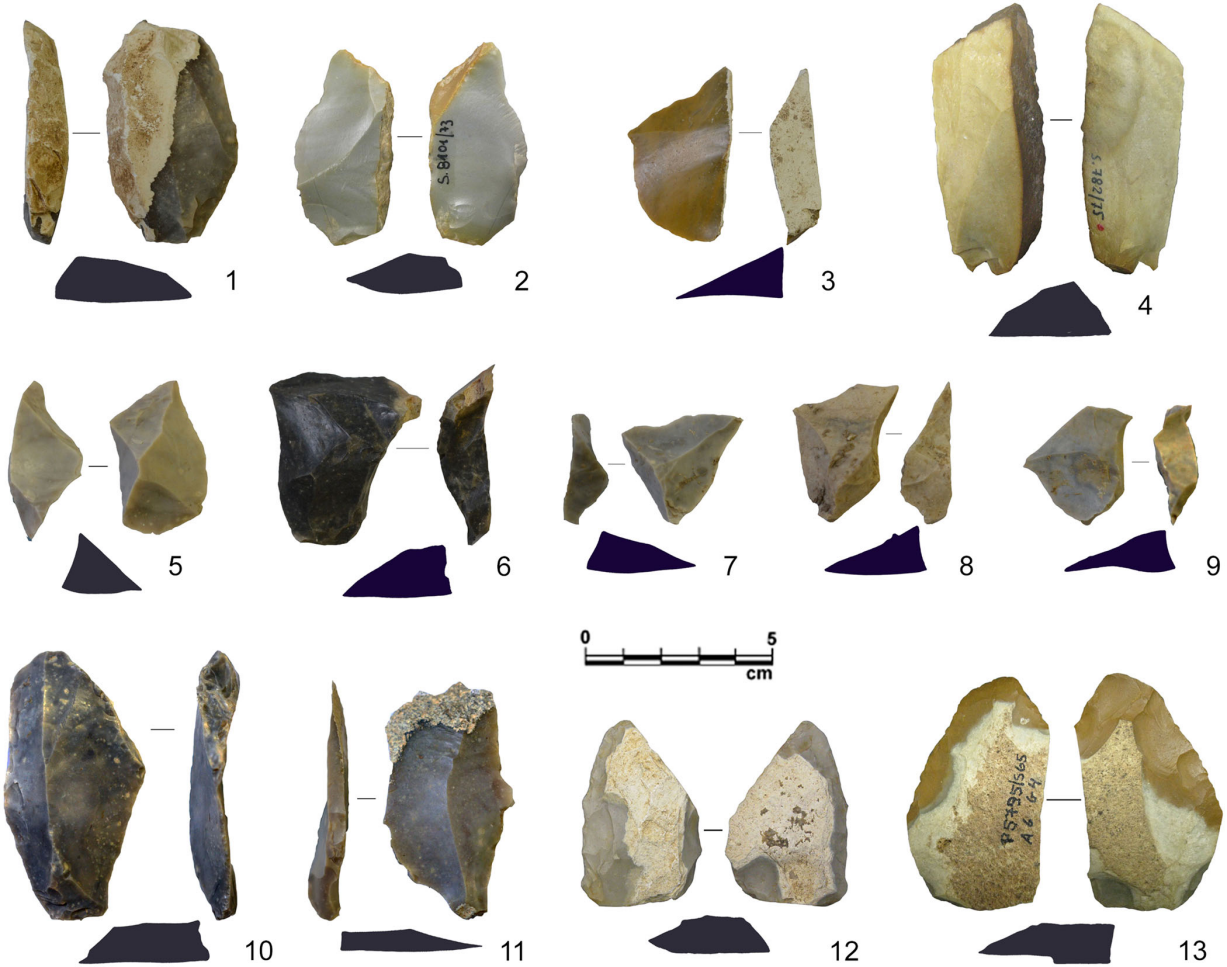
Naturally backed flakes (1–4), discoid core‐edge‐removal flakes (CERFs) and pseudo‐Levallois points (5–9), Levallois CERFs (10–11) and backed bifaces/*keilmessers* (12–13) from Fumane Cave A9 (3, 5–9) and A10–A11 (1, 10–11) and Sesselfelsgrotte G‐layers complex (2, 4, 12–13). *Source*: modified after Delpiano^27^.

The frequency of backed knives increases during the Middle Stone Age in eastern and southern Africa (e.g., Klasies River, Rose Cottage Cave). Here, backed knives are made on blades and flakes and are generally smaller than their Acheulean counterparts.^58^, ^59^, ^60^, ^61^ In parallel, all the main lithic reduction methods and volumetric concepts employed during the Lower and Middle Paleolithic of Eurasia (SSDA, Clactonian, discoidal, Quina, and Levallois) share, to varying degrees, the predetermined production of flakes equipped with a thick, blunt portion opposite a cutting edge as one of the main objectives (Figure 2). With the Quina Mousterian of western Europe, backed flakes with natural or flaked backs and a wide and asymmetrical triangular cross‐section is the main production objective (see^30^ for more details concerning the different types of “natural” backs). This is achieved through lateral and invasive detachments organized on subparallel and secant surfaces.^62^, ^63^, ^64^, ^65^, ^66^ These thick, asymmetric blanks were selected for the manufacture of Quina and half‐Quina scrapers. Thick and short flakes bearing natural or flaked back were also the objective of other Middle and Lower Paleolithic production methods, including the Clactonian/SSDA, the Tares‐type debitage, and the unipolar cobble flaking producing backed “orange‐wedges.”^67^, ^68^, ^69^

Discoidal reduction primarily involves bi‐pyramidal bifacial or unifacial cores on blocks and nodules or flakes which were knapped from the periphery with centripetal or chordal detachments. The latter help maintain the central convexity and the uninterrupted removal of flakes with a long flaked or natural back opposite a cutting edge (*débordant* flakes or core‐edge‐removal flakes—[CERF]) or a short lateral back opposite a point created by two convergent unipolar removals (i.e., pseudo‐Levallois points), which in some discoidal assemblages appear as the main production objective^35^, ^70^, ^71^, ^72^, ^73^ (Figure 2). In a similar way, recurrent Levallois modalities also produce CERFs, which could represent management products designed to maintain the core's lateral convexities as well as primary production objectives.^74^ Backed artifacts produced during Levallois reduction are not significantly different from the discoid ones except being generally thinner, longer with a more regular thickness and occurring in smaller numbers.^70^, ^71^, ^75^, ^76^

Backed bifacial tools also proliferate during the Eurasian Middle Paleolithic, becoming increasingly standardized, numerically more important and a common part of relatively uniform lithic assemblages of central and eastern Europe, namely the Micoquian or *Keilmessergruppen* techno‐complexes.^36^ The *keilmesser* (literally “wedged knife”), which both lent its named to this techno‐complex and often serves as a type fossil, does, however, occur in other geographical and chronological contexts.^77^, ^78^, ^79^ The *keilmesser* is an asymmetrical tool with a carefully bifacially retouched, sharp cutting edge, which can extend to a distally shaped point, opposite a usually unworked thick, often cortical prehensile part (Figure 2). Commonly considered to be hand‐held knives or scrapers, use‐wear analyzes have identified certain examples to have fulfilled multiple functions.^80^ Their particular morphology also afford *keilmesser* a highly strategic aspect, functioning as a core‐tool with an extended use‐live.^81^ In the shaping of backed bifaces, the back is often left unworked apart from extensive thinning which serves to (1) regularize the thickness and profile, (2) produce usable blanks from the lower surface, or (3) shape the double plano‐convex cross‐section which allows the tool to be reoriented.^82^, ^83^, ^84^

Finally, early blade production in the Levant, which is characteristic of several techno‐complexes, such as the Amudian facies of the Acheulo‐Yabrudian complex of the late Lower Paleolithic, is geared around the production of large quantities of naturally backed blades referred to as *lames débordants*.^85^, ^86^, ^87^, ^88^ The morphology of the thickest part of these blades is at times shaped by detachments from a previously knapped surface (flaked back or *dos de débitage*) or be cortical or neo‐cortical surfaces. In both cases, the back facilitates their immediate use without any need to adjust their ergonomic properties. With that said, in several Amudian contexts in the Near East these unmodified backed knives are accompanied by some of the first tools with retouched backs (see below).

## 4 THE EMERGENCE OF RETOUCHED BACKS: A PATCHY AND HETEROGENEOUS INNOVATION

### 4.1 The first retouched backs in Africa and the Levant

The first tools with retouched backs appear punctually in pre‐Upper Paleolithic and pre‐Late Stone Age context in Africa and the Levant. The early MSA Lupemban cultural complex possibly attests to one of the first instances of the systematic presence of retouched backed tools in the archeological record. The term “Lupemban” is, however, the subject of some debate, given its use as a “catch‐all” term for sometimes poorly contextualized sites and assemblages in central Africa, particularly in the Congo Basin. Lupemban assemblages are usually characterized by core‐axes, prepared core technologies aimed at the production of points, blades, and backed blades, as well as the emblematic elongated bifacial, lanceolate, and foliate points. It has been suggested that Lupemban assemblages potentially represent a late Middle Pleistocene rainforest and woodland‐adapted technology and toolkit.^32^, ^89^, ^90^, ^91^ Among the retouched tool component, small backed tools and *tranchets* are sometimes present in variable frequencies. These tools are referred to by a variety of terms; for example, *trapezoids, segments, microliths, truncated flakes and blades, crescents*.

**Figure 3:**
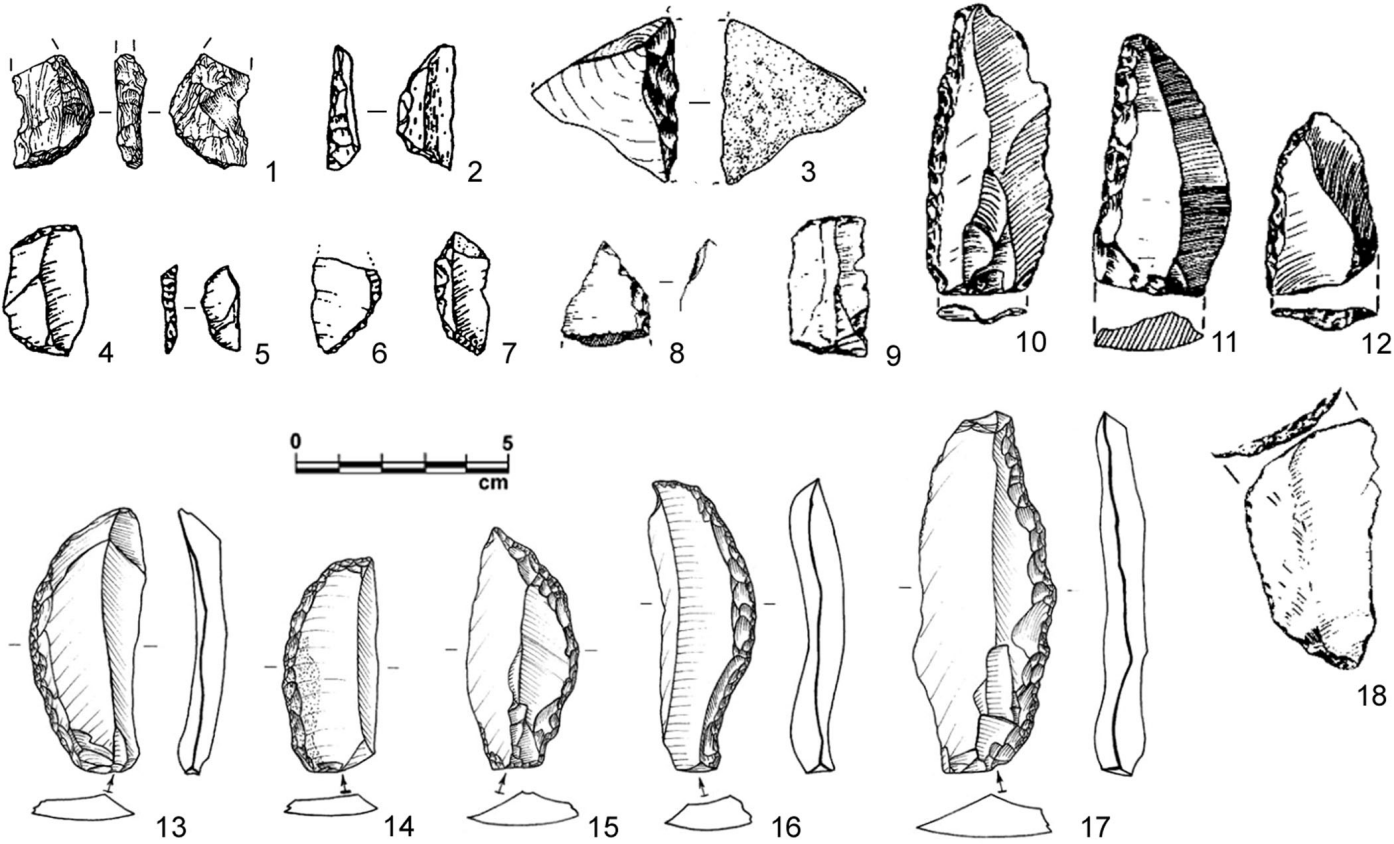
Retouched backed tools from African and Levantine Middle Pleistocene assemblages: Twin Rivers (1–2, 4–7), Sai Island (3, 8), Kalambo Falls (9–12, 18), Qesem Cave (13–17). *Source*: modified after Barkai et al.^86^; Barham^89^; Van Peer et al.^96^.

At Kalambo Falls, Zambia, a stratified “secondary” site currently lacking an absolute chronology, backed implements are present in the late Lupemban phase (Siszya), particularly backed flakes, backed blades, and possibly large backed *tranchets* together with truncated blanks, rare trapeziums and the more common presence of blade cores possibly reduced with a punch technique^92^ (Figure 3). A large broken trapezium (>40 mm) with a convex retouched back has also been interpreted as a *segment*. The small fraction of the Siszya industry was probably winnowed following postdepositional water action.^92^ The age of the later Lupemban is estimated to be between 266 and 170 ka (MIS 7) based on correlations with the U‐series dates from Twin Rivers, A Block. Twin Rivers is characterized by a microlithic industry with small backed segments (<30 mm) and trapezoidal tools made on backed or snapped flakes^89^ (Figure 3). After criticisms were raised regarding the reliability of the context, some “blocks” were considered unreliable while others (blocks A and F) recorded a sealed sequence, with the Lupemban dated by Uranium‐series to approximately 265 ka BP. At Broken Hill (Kabwe) Cave, Zambia, several backed flakes were recovered from a late Middle Pleistocene deposit dated to around 300 ky and well known following the discovery of *H. heidelbergensis*/*rhodesiensis* fossils.^93^, ^94^ However, no secure data concerning the context and the direct association between the human fossils and archeological material are available given the early date of the discoveries. Finally, the presence of small backed segments at Sai Island, Sudan (180–200 ky) is difficult to confirm based on the publication, which mentions triangular fragments with the possible presence of retouch and burin‐like impact fractures^95^, ^96^ (Figure 3).

As mentioned above, Qesem Cave, Israel, produced an Amudian industry potentially dated from MIS 11 to MIS 9‐7^97^ and characterized by the systematic production of blades by direct internal percussion and a variety of tools made on blades, including naturally backed knives (NBK) and blades with a curved retouched back.^85^ The latter includes eight backed blades (three with distal truncations), 17 curved backed blades, some pointed, and five backed flakes^86^ (Figure 3). Use‐wear analysis on the whole assemblage demonstrated the NBK were almost exclusively used for cutting soft materials, namely tissue and plant fibers. The three backed and truncated retouched blades, however, were possibly used for scraping tissues (*n* = 2) or cutting plants/wood (*n* = 1).^98^

Curved backed blades have also been reported from other Amudian assemblages, such as Abri Zumoffen,^99^ where A. Jelinek noted the appearance of a “truncated flake and blade” type in Tabun 48B, including distally retouched pieces.^100^ In the early Middle Paleolithic site of Misliya Cave (MIS7‐6), characterized by Levallois and blade assemblages, the prehensile areas appear to be modified by abrupt retouch, resulting in classic “backed knives.” The abrasion of cortical surfaces and dorsal ridges may also be linked to the preparation of tools to be used in the hand or for hafting or wrapping, a well‐attested behavior at Misliya, particularly for the Abu‐Sif points (elongated, laminar points) and small flakes.^101^

### 4.2 European Lower and Middle Paleolithic

Isolated cases of retouched backed tools are known from the European Lower Paleolithic. The “Acheulean backed knife” initially described by Bordes^25^ comprises a large back with moderately abrupt retouch often ending in a pseudo‐endscraper. These tools were reported from Saint‐Acheul, although is is unclear if the illustrated pieces bear simple or semi‐abrupt retouch. At Londigny (Charente, France), in addition to dozens of blanks with various morphologies as well as bifaces, both with natural backs, the natural backs of four pieces equally exhibit abrupt retouch opposite the cutting edge. In a single illustrated tool, the retouch concerns the mesial‐distal part of the blank, forming an irregular, convex‐denticulated edge with a semiabrupt angle. This modification could reflect either the installation of a prehensile zone or a denticulated active edge.^57^

**Figure 4:**
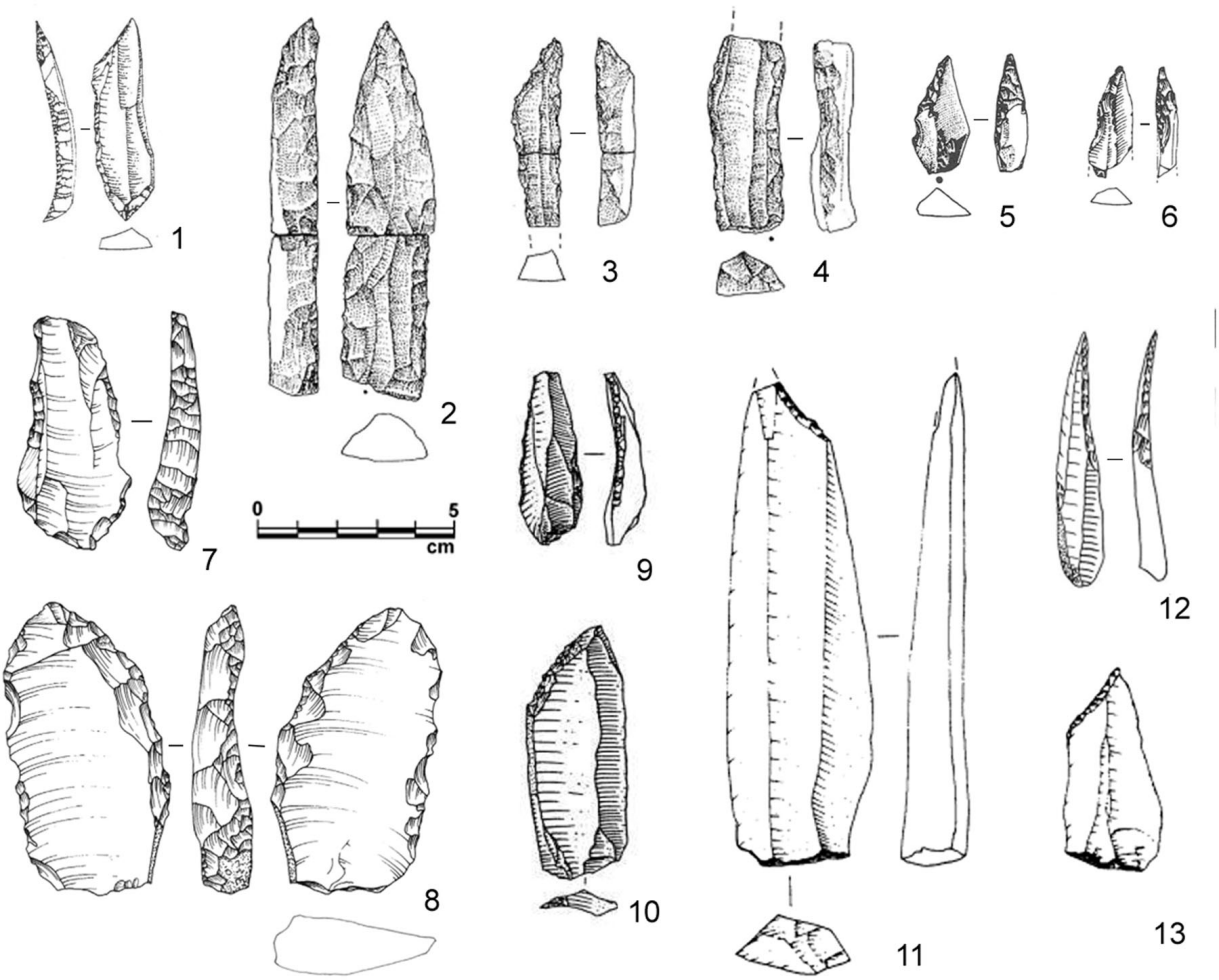
Retouched backed tools from ‘early’ Middle Paleolithic European assemblages (MIS 6‐5): Rheindahlen B1 (1), Wallertheim D (2–4), Tӧnchesberg 2B (5–6), Bisnik A6 (7–8), Riencourt‐lès‐Bapaume CA (9–10), San Francesco (11, 13) and Seclin (12). *Source*: modified after Klostermann and Thissen^104^; Conard and Adler^105^; Conard^106^; Cyrek et al.^107^; Vande Walle^108^; Bietti and Negrino,^109^; Tuffreau et al.^110^.

In western Eurasia, retouched backed tools first appear consistently among MIS 5 or slightly older Middle Paleolithic assemblages with a blade component, specifically on elongated blanks and usually together with a more varied toolkit including typically Upper Paleolithic tool‐types. The open‐air site of San Francesco, in Sanremo (Western Liguria, Italy), produced a highly distinctive industry with long blades detached from both prismatic and unidirectional bipolar Levallois cores. These blades exhibit either abrupt distal truncations (*n* = 16) or were transformed into backed knives (*n* = 30)^102^ (Figure 4). U‐Th/ESR dates and a new OSL dating program place the site to the end of the Middle Pleistocene, just before the last interglacial^103^ (Negrino, pers. comm).

Blade assemblages with backed tools are particularly common in northern European open‐air sites during MIS 5. In the Rhein Valley, the sites of Tönchesberg (2B horizon), Wallertheim (D horizon), and Rheindahlen (B1 horizon) produced assemblages that included a blade component and bladelets partly produced from burin‐like cores.^104^, ^106^ Some bladelets and points exhibit a double (bilateral) back, especially in the Wallertheim and Tönchesberg assemblages^105^, ^111^ (Figure 4). The faunal assemblages are dominated by horse, bovids, and deer, leading some to interpret the sites as hunting camps. The backed elements serving to arm hunting weapons is supported by recurrent damage on the tips. However, dedicated use‐wear studies are currently lacking.

The assemblage from Seclin (northern France), dated to MIS 5, produced a purely blade‐based assemblage, also described as “specialized Levallois.”^112^ Upper Paleolithic‐type tools are present, among which blades and bladelets with abrupt retouch on one edge, sometimes extending transversally onto the distal end^110^ (Figure 4). At Rocourt (Belgium), blades with abrupt marginal retouch are present together with burins and truncations. The material is preserved in a horizon containing reworked Eemian sands, placing it within a late phase of MIS 5. Other similar assemblages include Riencourt‐Le‐Bapaume (Somme Basin, France) dated to MIS 5a or MIS 5 which produced a handful of typical and atypical backed knives on flakes and blades^108^ (Figure 4). Based on stratigraphic and paleoenvironmental data, as well as thermoluminescence dating, all these sites record sequences from the last Interglacial to the first part of the Weichselian glaciation, where assemblages with backed tools are generally positioned. The appearance of these new toolkits from blade assemblages raises several interesting questions; among these, the possibility of the backing of blades without natural backs serving to facilitate their use and the emergence of a technical adaptation that will come to characterize nearly all subsequent blade assemblages.^113^

In eastern Europe, a handful of retouched backed knives on unipolar Levallois flakes and blades have been reported from Bisnik Cave (A6 assemblage) and dated to MIS 7. However, the material exhibits detachments on both the upper and lower surface, possibly due to postdepositional reworking, making it difficult to determine the origin of the abrupt retouch on the back^107^ (Figure 4). Backed knives are also present in the Dniestr Basin (Velykyi Glybochok), where either a natural or an artificial back is present mainly on blades and Levallois flakes.^114^

### 4.3 The European Late Middle Paleolithic

Retouched backed artifacts have and continue to be problematic in the definition and characterization of the Late Middle Paleolithic techno‐complexes in western Europe, as a whole, and southwestern France in particular. Backed knives, originally a defining feature of Bordes' Mousterian of Acheulean Tradition Type B,^33^, ^115^ in fact appear in variable numbers and associated with multiple flake production systems in the late Mousterian of MIS 3.^47^, ^72^, ^116^ Confusion surrounding the relevance of these tools for Mousterian industrial variability is partly due to the fact that many of the key assemblages containing large numbers of these tools were excavated years ago, involving nonmodern recovery methods and little stratigraphic control. A shift to an emphasis on technologically based definitions of Middle Paleolithic techno‐complexes has recently been proposed^117^, ^118^ and recent re‐evaluations of key “MTA‐B” assemblages^72^ have also shown the continued use of the moniker “MTA” to be misleading, collapsing a significant degree of technological diversity into a single typological “facies” which should be abandoned.^119^

**Figure 5:**
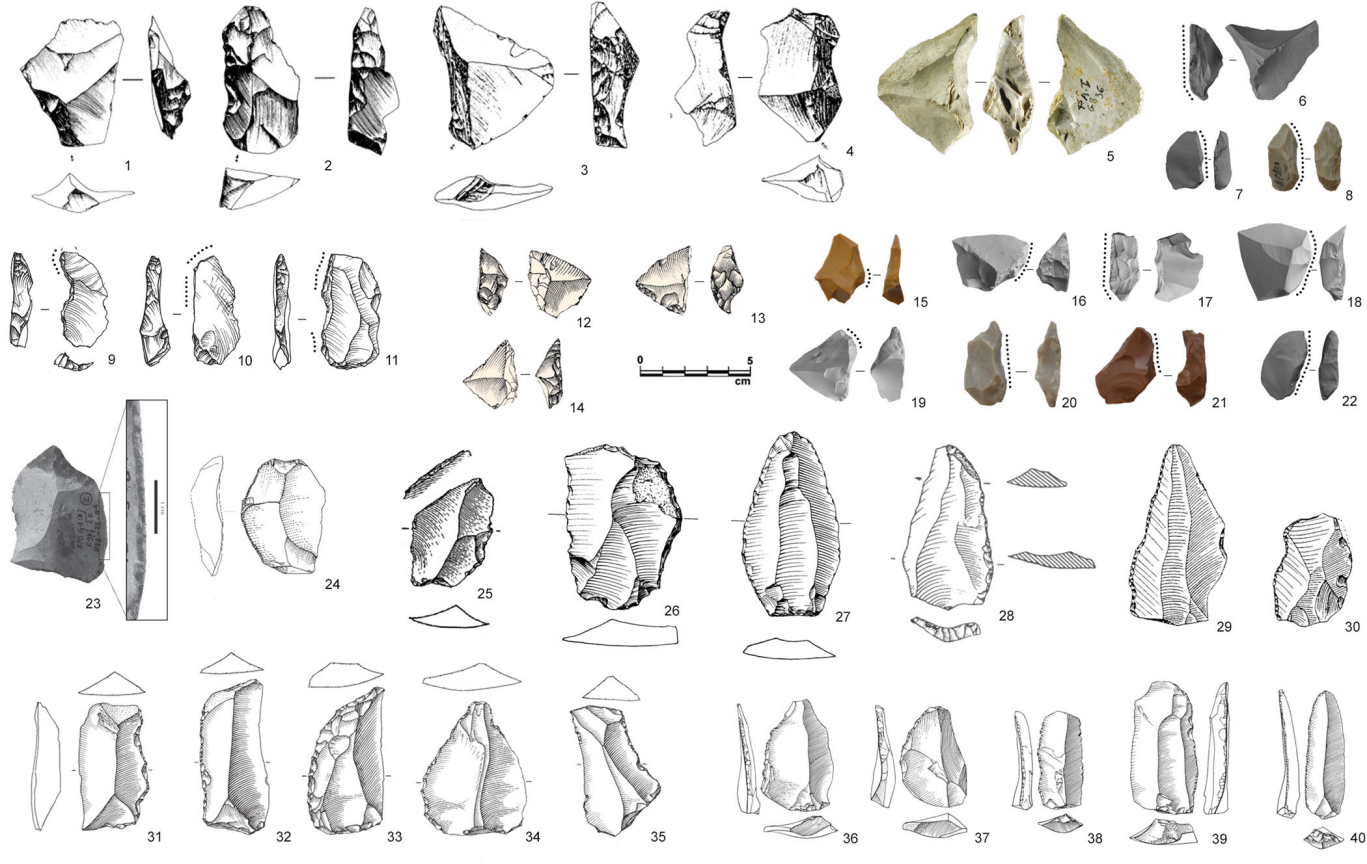
Retouched backed tools from late Middle Paleolithic assemblages in Europe (MIS 3), including Discoid (1–24), Levallois (25–28) and unipolar (29–40) blanks (23–28): Beauvais (1–4), Pech de l'Azé I 6–7 (5, 36–40), Fumane A9 (6–8, 15–22), Ormesson (9–11), Mandrin D (12–14), Saint‐Césaire Egpf (23–24), La Rouquette B (25), Grotte d'Engihoul (26–27), Plateau Cabrol (28), Abri Chadourne A (29–30), La Rochette 7 (31–35). *Source*: modified after Loch and Swinnen^124^; Soressi^121^; Delpiano et al.^50^; Slimak et al.^125^; Bodu et al.^126^; Thiebaut et al.,^127^; Duran and Tavoso^128^; Turq^30^; Ulrix‐Closet^129^; Bordes^115^. Photo of piece n°5 is courtesy of P. Jugie, Musée national de Préhistoire.

With that said, backed tools do exist in French Middle Paleolithic assemblages, mostly in the south‐western regions: La Rochette layer 7, Pech de l'Azé I layers 6‐7, Pech de l'Azé IV layers F1‐F2, Gare de Couze, La Métiaire a Belcayre layer C1, La Grande Roche de Quincay, Goderville^27^, ^47^, ^120^, ^121^, ^122^, ^123^ (Figure 5). While true that in these assemblages backed tools can reach between 10% and 30% of the retouched assemblage, most were excavated long ago and recovery biases influencing tool counts can be suspected.

Backed knives display a fair degree of variability in terms of retouch type, intensity, and extent. Retouch is almost always direct on one edge and is usually marginal and abrupt or semi‐abrupt, with some pieces displaying invasive retouch. Retouch usually but not systematically produces a slightly convex to markedly curved back, sometimes ending in a robust trihedral point (Figure 5). Retouch can occasionally continue onto the distal portion of a blank, creating a sort of “pseudo‐endscraper.” Retouch is designed to blunt the portion of the tool opposite an active cutting edge, creating a prehensile area.

While backed blanks are reported as usually being on elongated, thick, symmetrical flakes with variable widths and cortex, this equally may reflect recovery biases linked to early excavations. In assemblages that feature a substantial discoidal component, which coexists with unipolar reduction or is mixed due to excavation bias, unipolar flakes are usually but not uniquely selected for the production of backed knives. The selection of the most elongated blanks for tool manufacture is recorded in two contexts: La Rochette layer 7 and Pech de l'Aze I layers 6 and 7^27^, ^131^ (Figure 5).

Another recurrent aspect of the Western European Late Middle Paleolithic is the presence of a varied set of mostly “atypical” backed tools in what are nearly exclusively discoidal assemblages (Figure 5). Layer A9 of Fumane Cave, in north‐eastern Italy, is an instructive example. This discoidal assemblage, dated to around 47.6 ky BP, produced more than 30 typical discoidal products, namely pseudo‐Levallois points and CERFs, that exhibit abrupt retouch on a flaked or cortical back which almost certainly was the nonactive portion of the tool.^50^ This “backed back” was installed using either direct percussion or bipolar percussion on an anvil, sometimes subsequently regularized by simple abrasion (Figure 1). This additional “backing” of an already abrupt edge likely served to shape or straighten a pre‐existing thick prehensile portion, blunt a dihedral protuberance or increase the angle with the ventral surface (between 72° and 85° on average) or adjust the thickness in profile.^50^ These modifications also potentially allowed the tool to be more easily used in the hand or inserted in a haft. Numerous small, retouched pseudo‐Levallois points were detached from cores‐on‐flakes using a “Kombewa‐type” technology^130^ typical of discoidal industries.^73^, ^131^ The morphological and technological diversity of the backed tool assemblage from Fumane associated with a core‐on‐flake reduction strategy is consistent with these tools being modified to satisfy immediate functional needs as part of local to regional mobility systems. Similar technological patterns have been reported from several caves and rock‐shelters in south‐western France, including Combe Grenal (Layer 1), La Quina Amont (Layers 4, 6a, and 6c), St. Césaire (Layer Egpf), and especially Le Moustier (layer H), where the latter also producing a significant number of retouched pseudo‐Levallois points.^72^, ^117^, ^127^, ^128^, ^129^, ^130^, ^131^, ^132^, ^133^, ^134^

Coeval assemblages from northern France (Beauvais and Les Bossats in Ormesson) confirm this behavior and point to the association of triangular elements with a robust point or a spine opposite a blunted back that can be straight, curved in the distal portion or concave in the mesial part^124^, ^126^, ^135^ (Figure 5). In south‐eastern France, the same association and chronology (with almost identical form and modifications) is reported in the Post‐Neronian layers (5 to 1) of Mandrin Cave^125^ (Figure 5). Pseudo‐Levallois points (*n* = 55) seem to be preferentially selected for a direct abrupt retouch of the back, referred to by the authors as a form of “truncation,” with preliminary functional analysis of the late Mousterian pseudo‐Levallois points suggested diagnostic impact fractures on four truncated and nine nontruncated pieces.^136^

Despite the numerous occurrences and long chronology of Levallois technologies during the Middle Paleolithic, it is interesting to note that Levallois assemblages generally lack retouched backed tools. However, when these tool types are present, they are usually manufactured on elongated and unipolar blanks, as at, for example, La Folie, La Roquette, and La Plane^30^, ^128^, ^137^ (Figure 5). Assemblages featuring smaller numbers of what are mainly atypical‐backed knives are known from France, Belgium, Spain, and Italy.^30^, ^47^, ^64^, ^81^, ^113^, ^115^, ^129^, ^138^, ^139^, ^140^, ^141^ These tools reflect highly diverse technical behaviors from blank selection to the location, extent, and intensity of retouch. This lack of standardization would be consistent with a relatively expedient toolkit and a degree of opportunism in the application of backing. With that said, this issue requires a dedicated, multi‐scalar approach combining techno‐economic analyzes with use‐wear data. Moreover, their inconsistent geographical and chronological distribution could equally reflect research biases and the fact that the little attention is commonly given to these tools.

### 4.4 Differences in Late Neanderthals backing of tools: Two assemblages compared

Deliberate modifications of flake edges through abrupt retouch increases in European late Mousterian assemblages, particularly in discoidal lithic technocomplexes. In these contexts, retouching primarily concern blanks already featuring backs in the form of core‐edge flakes and pseudo‐Levallois points. At Fumane, in northern Italy, the lithic assemblage from stratigraphic unit A9 includes at least 42 artifacts that have been modified by either complete (type 1) or partial (type 2) direct retouch. Typical backed knives are also commonly associated with other types of blanks in the late Middle Paleolithic, namely elongated flakes produced following a non‐Levallois unipolar blade‐like technology that have a symmetrical triangular section and differ significantly from the morphology of typical discoidal backed products. One of the best examples is the aforementioned site of La Rochette level 7 (Delporte excavations) which produced 73 typical and atypical backed knives. These two assemblages allow the variable application of backing by late Neanderthal groups to be explored.

**Figure 6:**
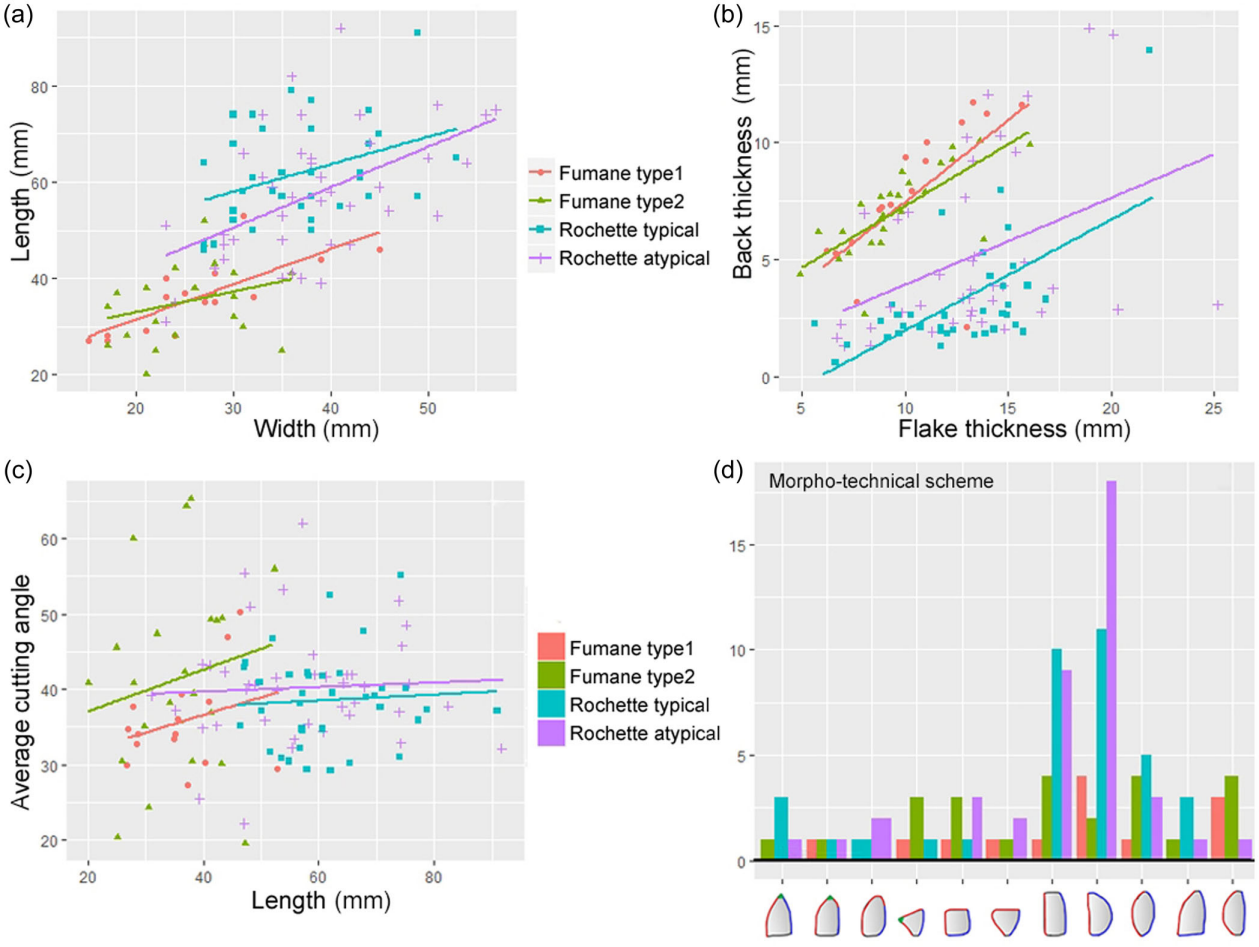
Morpho‐technical and metrical comparison of backed tools from Fumane A9 (type 1: complete retouch; type 2: partial retouch) and La Rochette layer 7 (typical and atypical backed knives). Length/Width ratio showing a tendency towards elongation (a); ratio between the thickness of the blank and thickness measured on the back, showing a thin back on La Rochette tools compared to tool thickness (b); development of the mean cutting angle in respect to the tool size (c) and diversity of morpho‐technical scheme (d).

The Fumane tools are consistently less laminar and characterized by much thicker backed edges, even if flakes have similar maximum thicknesses. With the La Rochette backed knives, the maximum thickness is towards the center of the blank, while discoidal products are both less standardized with the maximum thickness of the product being the backed edge (i.e., the detached edge of the core) (Figure 6). Given the differences in the blank selection, the modification/retouching of the back in both assemblages probably reflects different goals. At Fumane, the blunting of the prehensile edge is presumably intended to facilitate the application of pressure on the backed edge via a manual grip or handle. The retouching was designed to either render an excessively thin edge more robust or blunt a sharp or irregular edge that did not allow a direct grip. On the other hand, with the La Rochette tools, the steep retouch provides a means to create a passive prehensile edge opposite the sharp active edge of the symmetrical elongated blanks. Unlike the Fumane examples, the retouch on the La Rochette tools is relatively standardized, taking the form of a continuous, marginal retouch along one edge. Moreover, the backed knives can be separated into two sets of tools with different techno‐functional aspects: elongated tools with a straight cutting edge opposite a convex back and elongated tools with two, parallel, straight edges (a passive retouched edge opposite the active cutting edge). A certain standardization is evident in the cutting angle of both sets, generally figuring around 40° regardless of tool size and blank type (Figure 6).

At La Rochette, the standardization in technical investment (type, location of retouch, and blank choice) and resulting tool morphology (consistent active edge angles and recurrent techno‐functional aspects) suggests the implication of a particular preconceived notion of how to make blanks functional. Conversely, the high degree of variability in these same characteristics among the Fumane backed tools suggests an expedient solution to particular ecological circumstances and functional needs^27^ (Figure 6). These differences would therefore reflect “function driven tools,” in the case of Fumane, whose production fulfills specific functional needs, and “concept‐based tools” with potentially preconceived forms in the case of La Rochette.

## 5 DISCUSSION

### 5.1 Naturally backed artifacts from the Lower to Middle Paleolithic

The earliest stone tools appear to have primarily served as “knives” or “axes” to separate material via slicing or cleaving.^142^ These hand‐held tools require the necessary anatomical apparatus and manual dexterity to enact relatively concise movements, involving precision grips, wrist rotation, and opposable thumbs, traits that likely predate the emergence of the first stone tools. Multiple anatomical features (short fingers compared to the thumb, less curved phalanges, a long robust thumb, a specialized wrist, and fingertips with broad fleshy pads underlain by wide apical tufts of bone) distinguish humans from nonhuman primates. These aspects afford a stable and robust precision grip that increases stability when gripping small tools.^4^, ^143^, ^144^, ^145^

The use of these tools involves a passive element that serves as either a prehensile or receptive zone in the tool‐human system.^45^ With the earliest stone tools, this portion is usually unprepared and not predetermined, with ergonomic aspects and potential tool grasps influenced by both tool shape and size. Recent experiments have, in fact, shown that stone tools are manipulated in a similar fashion by different individuals independent of their morphology, suggesting this would be equally the case throughout the course of human evolution.^55^ The use of handheld tools equally depends on the location of their center of mass, in the sense that well‐balanced tools reduce stress and potential injury to the hands, arms, and shoulders.^146^ The handling of tools would naturally focus on the thicker, nonactive part, which is usually unmodified. In the case of simple flake production, the platform or a naturally thicker lateral part of the tool could be included in this arrangement, with the thumb stretched onto one surface, and the other fingers (mainly the index and middle finger) on the opposed surface or partially accommodated along the noncutting edge. According to Feix et al.,^147^, ^148^ this simple configuration is consistent with a power grasp, combining palm opposition and thumb adduction, and potentially including an extended index finger and different degrees of flexion.^55^ As a general rule, the location of the prehensile element of cutting tools is above all conditioned by the location of easiest part of the tool to grasp in relation to exerting force with the active part used to transform the material.

As with all technical actions, increased ease of use and a more efficient grip combine to prevent damage to the hand while, at the same time, improving tool performance. Experimental studies have suggested that the ergonomic features of stone tools, namely their morphology, morphometry, and weight, potentially influence tool grasping in terms of finger flexion, type of handling, grip force, and overall comfort.^149^, ^150^ As mentioned above, stone tools equipped with natural or modified backs are a common feature of both Lower and Middle Paleolithic assemblages. These recurrent tool forms raise a series of related issues, in particular, the fact that the primary goal of the knapper is not the production of a specific tool “type,” but rather the fulfillment of a basic need that drives tool production and use. In this sense, the recurrent production of a tool “type” or “morphology” speaks to its inherent effectiveness or efficiency in responding to the needs of human groups. Once this idea took hold, it slowly evolved both in terms of tool morphology and techno‐functional specialization. As such, retracing the functionality of unretouched backed tools may help elucidate the factors driving the emergence of intentional “backing.”

Current use‐wear evidence shows cutting and slicing to be the most common activities recorded on Lower Paleolithic stone tools, including artifacts with cortical “backs.” At Koobi Fora, Kenya, several flakes with cortical surface opposite a cutting‐edge exhibit wear referable to cutting motions, primarily involving meat or highly siliceous stems of grasses.^151^, ^152^ At Olduvai Gorge (Tanzania), Kada Gona (Ethiopia), and Kanjera (Kenya), use‐wear analyzes have generated similar evidence for quartz and basalt flakes being used to scrape wood, cut fresh meat and hides, or process vegetal fibers.^153^, ^154^ In Acheulean assemblages, use‐wear analyzes have almost exclusively focused on bifacial tools, at the expense of other retouched tools, including backed knives. While Lower Paleolithic bifaces fulfilled multiple functions, including processing carcasses and working wood in addition to other more heavy‐duty tasks,^155^, ^156^ the less morphologically variable backed knives point to more specialized functions. Backed knives from Wonderboom, South Africa, dated to between 800 and 500 ka, were expedient, short‐use artifacts with an ergonomic form designed to optimize meat acquisition and butchery.^53^ Similarly, Amudian naturally backed knives from Qesem Cave, manufactured on laminar blanks, were almost exclusively used for cutting rather than scraping activities, namely the removal of meat and connective tissues from bone. Naturally backed knives from Nesher Ramla (MIS 5‐6) were mostly used to work harder materials: interestingly, blade blanks were preferred for cutting, while flakes were used for scraping.^157^ Traces are usually recorded along the sharp edge opposite the natural back and less often, on the distal and proximal ends. Retouched edges were reported as being relatively inefficient for defleshing carcasses, as they tended to require the application of more force to cut compared to tools with thin, unretouched edges. However, the same study did report that if a carcass is held steady, a “retouched butchering knife” was most effective for slicing off large pieces of meat.^157^

In terms of the Middle Paleolithic knapping strategies, the functional effectiveness of backed artifacts (core‐edge flakes and pseudo‐Levallois points) from Levallois and discoidal reduction in the late Mousterian of Fumane Cave was addressed via a techno‐morphological analysis.^71^ The high cutting effectiveness of Levallois flakes is due to the low edge angles relative to overall tool size and the regularity of the bevel. Moreover, the relatively regular thickness of the nonactive portion allows these flakes to be better grasped in the hand, affording a more efficient transfer of force while also rendering them adaptable to different hafting arrangements. Backed products from the Levallois system can also be used unhafted or hafted for general butchering activities,^80^, ^158^, ^159^ although cutting and sawing has also been recorded.^160^ The morphology of backed artifacts produced in discoidal reduction, on the other hand, is more variable, possibly related to the need for tools to respond to a wider range of actions. This more flexible functionality reflected in the use of the edges, tip, or convexity created by the conjunction of the flakes negatives on the dorsal face.^71^ Discoidal backed flakes are both efficient for use unhafted, especially when equipped with a convex back, or can be adapted to hafting after the reconfiguration or modification of the back.^50^ Available use‐wear data is consistent with high functional versatility of discoidal blanks, including artifacts that show some degree of standardization such as pseudo‐Levallois points.^137^, ^161^, ^162^ A notable difference between the two reduction methods is the near systematic presence of backed artifacts produced by discoidal reduction, which is potentially linked to the need for increased transfer of force during use when hand‐held.

Moreover, the functionality of unretouched backed bifaces, such as keilmesser, does not differ fundamentally from that of backed flakes, due to the asymmetric plano/convex shaping of the cutting‐edge.^36^ Keilmesser were mainly used unhafted for a variety of activities.^80^, ^163^ Their continuous bifacial shaping/resharpening was also a potential source of smaller flakes with complementary functional attributes.^81^, ^164^ Shaped‐backed tools generally have long use‐lives due to the normally substantial volume of exploitable raw material allowing them to function as core tools.^165^ A similar concept is equally evident in Quina scrapers, which are often manufactured on thick flakes equipped with natural or knapped back that are heavily reduced and recycled.^64^, ^166^ Backed tools in the Eurasian Middle Paleolithic appear to be generally connected by the absence of task specialization, which is related, on the contrary, to a high degree of versatility and capacity to fulfill multiple purposes and functions.

### 5.2 The invention(s) of backing: Functional advantages for handling and precision tasks

The appearance of retouched backed tools in many Middle Paleolithic assemblages could have several important repercussions for improving efficiency during daily activities. Retouched backs naturally afford safer manual prehension by dulling and remodeling the nonactive edge of the tool. Use‐wear data from typical backed knives recovered from the Mousterian site of Pech‐de‐l'Azé I (France), manufactured on elongated flakes with two roughly parallel edges and symmetrical cross‐section, have been associated with manual prehension.^167^ This backing concept was likely designed to avoid the significant time and energy investment and need for additional raw materials for the creation and maintenance of a haft. While normally the back is installed on one lateral edge by direct, abrupt retouch, this modification sometimes encompasses (or uniquely concerns) the distal portion of the tool, leading them to be confused for genuine “end scrapers.”

Targeted experiments have equally demonstrated the utility of retouching what are already abrupt or natural backs.^50^ This additional backing modification was observed in Mousterian assemblages characterized by discoidal technology and blanks, namely core‐edge flakes and pseudo‐Levallois points, usually equipped with a “dos de débitage”^35^ or “dos de negative.” In these cases, using a flake with an unmodified back could be uncomfortable either due to its shape or the presence of a sharp dihedral surface. This would be particularly the case when applying pressure with the fingers during tool use. Therefore, blunting by means of retouching helps protect either fingers or fibers used to lash the artifacts to a handle. Specific modifications of the back therefore help to improve the grip and, consequently, the functional effectiveness of tools during cutting or scraping activities. Such modifications are particularly effective to increase grip when performing tasks where hands get covered in grease or fatty tissue during, for example, carcass processing.^50^ Moreover, the configuration of the back may have served to modify longitudinal profiles, which is equally relevant for functional reasons. Convex backs proved to be highly efficient for tool manipulation when manual grasping involves the accommodation of one finger on the back of the tool, such as in the precision grasp with pad or side opposition and particularly in the palmar/tip pinch or the writing tripod variants.^147^ This kind of grasping is effective when moderate force is required, while in tasks that require greater strength and a very firm grips, a power grasp with palm opposition is necessary. In this configuration, fingers are placed on the two surfaces of the flake with the thumb inwards, therefore avoiding direct contact with the back, which consequently does not require a specific shape.^50^

The emergence of the backing of stone tools could be possibly related to selective pressure linked to the increased use of precision grasps and a reduction in tool size.^40^ Pinch/tripod dynamic grasps are needed when small tools are manipulated in the absence of wraps or hafts, which in turn implies the finger(s) being supported on the back of the tool. This miniaturization of stone tools is, however, heavily dependent on the geographical and chronological context.^40^ While specific trajectories for small tool production and use (<30 mm) have been reported for early Acheulean assemblages at Olduvai Gorge, no specific conclusions were drawn concerning the need for a specific degree of manual dexterity necessary to them.^52^

Small tools are present in Lower Paleolithic assemblages across Europe. Among the earliest assemblages characterized by small tools is Isernia La Pineta (Italy), dated to MIS 15, where small unretouched flakes were used for processing soft and soft‐to‐medium hard materials (meat, fresh hide, and animal tissues).^168^ The efficiency of small retouched and unretouched flakes for butchery activities on large herbivores carcasses has been demonstrated in multiple experimental contexts and recorded at several other European Lower Paleolithic contexts.^169^ At Ficoncella (Latium, Italy), a MIS 13 site with elephant remains, one of the main productions objectives are very small tools with a “narrow cutting edge with a very open angle, a spine and a lateral cortical back.”^170^ The presence of small flakes in association with elephant remains is, in fact, a recurrent pattern at Lower Paleolithic sites throughout the Middle Pleistocene in Europe, Africa, and the Levant.^171^, ^172^, ^173^, ^174^, ^175^, ^176^ In one of these, Gesher Benot Ya'aqov, in the Levant, a proximal modification on small flakes has been interpreted as a preparation for hafting, but no functional analyzes have been performed.^177^

Throughout Prehistory, the production and use of small tools is considerably more common than the use of the more visible large stone tools, as extensively addressed in a recent review by Pargeter and Shea.^40^ However, this process of miniaturization only really begins with the Middle Paleolithic and Middle Stone Age, which equally sees the emergence of what are sometimes relatively standardized “backed” tools as a regular feature of stone tool assemblages. These tools may also mark a bio‐cultural shift, as they appear in tandem with the appearance of archaic *Homo sapiens* and *Homo neanderthalensis*. The small size of the modified backed tools from Fumane, for instance (25–35 mm), generally smaller than scrapers (which are on average between 40 and 45 mm), together with the length of the transformative area on the tool and the limited extension of the use‐wear traces along the edge, would indicate that these artifacts could be interpreted as specialized tools for precision cutting.^50^ Increasing evidence highlighting the capabilities of Neanderthals to perform precision grips relying on the thumb and the index finger^178^, ^179^ potentially explains the sparsity of evidence for hafted tools during this period. This would equally be consistent with experimental evidence that suggests that most Mousterian backed tools associated with discoidal technologies were likely used in the hand.

### 5.3 Backing and hafting: Interrelated or alternative techniques?

**Figure 7:**
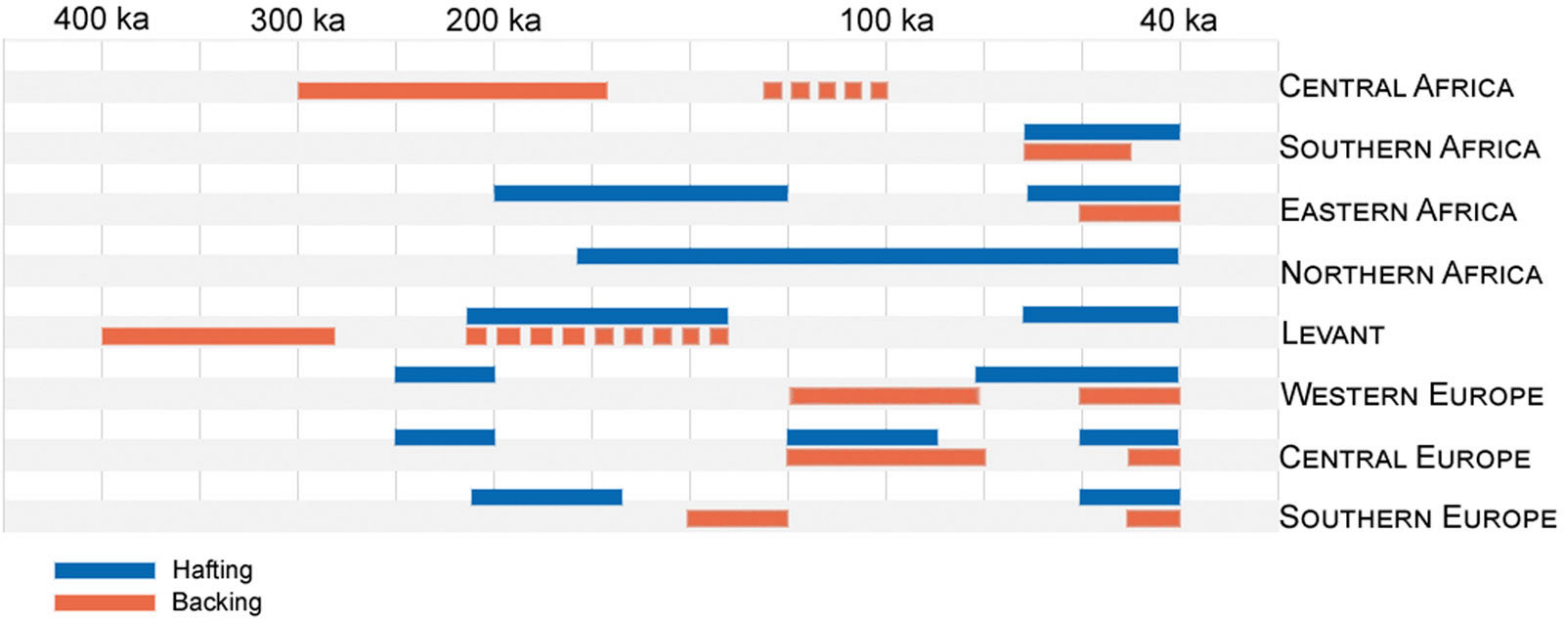
Summary diagram for the appearance and timing of backing and hafting during the Middle Palaeolithic (MP) and Middle Stone Age (MSA) from ~400 to 40 ka in different regions of Africa, Europe, and the Levant.

Most investigated applications of backing are related to the design and arrangement of composite tools despite evidence for the hafting of unmodified or nonbacked implements. The emergence of hafted cutting tools has also been considered as an important step in the evolution of prehistoric technologies. Hafting requires a complex array of knowledge for transforming what is often multiple materials and complex procedures required to produce effective composite tools. The earliest cases of hafted stone tools are documented in the Middle Pleistocene, rapidly becoming more common in the Late Middle Pleistocene, both in Eurasian and African assemblages^95^, ^180^, ^181^ (Figure 7). Hafting as a means to improve the effectiveness of stone tools was employed by both Neanderthals and early *H. sapiens*; however, given the perishable nature of the likely haft components, direct evidence remains relatively rare compared to later periods. For the Middle Paleolithic hafting equally included the developed of adhesive technologies, for which the current earliest evidence comes from the MIS 6 site of Campitello Quarry on the Italian peninsula.^181^ Plant‐derived adhesives, such as pitch produced from birch bark, conifer resin, and beeswax, were used at the MIS 5 site of Inder‐Altdorf,^182^ as well as at the MIS 3 sites of Konigsaue, Zandmotor North Sea beach, Fossellone, and Sant'Agostino Cave.^183^, ^184^, ^185^ Bitumen was used for the same purpose in the Near East at least as early as MIS 4^186^, ^187^ and in the late Middle Paleolithic of Eastern Europe^188^; compounds of bitumen mixed with ocher were possibly used to render hand‐held tools more efficient in the Middle Paleolithic of Le Moustier (Upper Shelter), but secure contextual and analytical data are unfortunately lacking.^189^ In these cases, hafted tools show significant variability, including retouched pieces and unmodified flakes, points, and blades.

Significantly more indirect evidence for hafting is available in the form of use‐wear analysis.^190^, ^191^ An exceptional case of hafting by Neanderthal groups comes from the early Middle Paleolithic (MIS 7‐6) site of Biache‐St‐Vaast.^180^ This open‐air site produced at least 50 pieces, Levallois flakes, Mousterian points, different kinds of side‐scrapers, hafted distally and used as butchering knives, woodworking tools, and potentially as a spear point. Developed and varied hafting technologies were therefore already present in the early stages of the Middle Paleolithic. Hafted stone tools are sporadically present in other Levallois assemblages throughout the Middle Paleolithic and in multiple regions: Levallois points, blades, and flakes in the Levant,^186^, ^187^, ^192^ Mousterian or Keilmessergruppe assemblages with Levallois blanks in central, western, and southern Europe,^80^, ^158^, ^160^, ^184^, ^189^, ^193^ and diverse early to late MSA assemblages with Nubian Levallois methods in northeast Africa.^95^

Levallois blanks share some morphological features that make them more easily hafted compared to discoidal, Quina and elongated blanks, these include regular profiles and generally thinner cross sections.^71^ These shared features of Levallois products likely goes some way in explaining the emergence of hafting in association with this concept.^46^, ^180^, ^194^ While discoidal flakes are adaptable to hafting after reconfiguration of the back through retouching and thinning,^50^ evidence for hafting in these assemblages remains rare. If for the majority of Middle Paleolithic tools (butchering knives, scrapers for woodworking), hafting is not necessarily required why is it applied? Hafting involves a considerable amount of technical investment, the collection of raw materials, and time for the application of diverse manufacturing technologies, including adhesives, wraps, or organic handles. In fact, the time and energy exerted in hafting a tool far outweighs the time needed to produce it. Nonetheless, the benefits of hafting tools are evident^32^, ^43^, ^89^: (1) they are easier to hold; (2) protect the fingers from the sharp edge of the stone tool and thus injuries; (3) they render tools more effective by increasing the amount of force applied with less effort; (4) from an evolutionary perspective, hafted tools acted as a “performance equalizer,” reducing selective pressures related to biometric variables.^44^ Last but not least, composite tools open doors to new hunting innovations, namely mechanically delivered projectiles in the form of stone tipped spears or arrows.^195^

Several of these tools are already evident among the earliest instances of hafting technology, such as the spear points from Bianche‐St‐Vast^180^ and the proposed hafted points from Kathu Pan, potentially dating to ~500–300 ka.^14^ This would suggest that hafting developed from an accumulated knowledge base combining multiple technological elements. In this “evolutionary stage,” the hafting of tools is a prerequisite for their use. Unsurprisingly, the clear benefits of hafting for tool use would reinforce its simultaneously testing and applications for other functions in the early stages of the Middle Paleolithic and Middle Stone Age.

During this phase, only a small part of hafted lithic artifacts shows evidence for deliberate modifications to facilitate the insertion of the tool in a handle, these include the thinning of an edge or base on one or both the surfaces^196^, ^197^, ^198^ or the creation of a tang, which is frequently linked with hafting in northern Africa MSA.^95^, ^199^ Another potential hafting modification is the application of abrupt retouch (backing) on the section of the tools in contact with the haft, which can be combined with thinning.^50^ However, even if hafting and backing technologies may be correlated, they initially emerge and developed independently of each other of a substantial period of time and, in the earliest cases of hafting, may have been used alternately. The main benefits of hafting (protection, easy of grip, and greater efficiency) are also shared by backing, as was experimentally shown in the use of backed, hand‐held flakes from discoidal reduction.^50^

**Figure 8:**
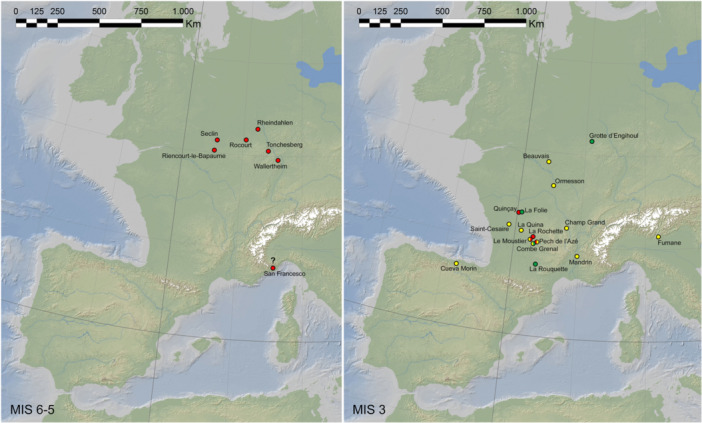
Map illustrating the distribution of the main stratified sites attesting to the presence of retouched backed tools in the Middle Paleolithic of Europe, distinguished by chronology (MIS 6‐5 on the left, MIS 3 on the right) and technology of lithic assemblage (red: laminar; yellow: discoid; green: Levallois).

Notwithstanding some bias linked to the uneven application of functional studies and the sporadic attention paid to Middle Paleolithic‐backed tools, some trends are nevertheless evident. In certain contexts, (i.e., Levallois assemblages), hafting is much more frequent than backing, which occurs only sporadically. In other cases (i.e., discoidal and Middle Paleolithic blade assemblages) backing is frequent and evidence for hafting is extremely rare (Figure 8). With the increasingly widespread use of hafted tools during the Middle Pleistocene, Levallois products appear to be the most suitable for this purpose, even without specific technical modification to adapt the morphology of less regular blanks.

The invention of backing therefore seems to be hypothetically related to hafting only indirectly, as an alternative solution to improve the performance of retouched tools and flakes that are less suited to complex hafting arrangements. Among the very first cases of backing are the Qesem Cave backed knives, which already reflect a fully structured and standardized application of the techniques for the manipulation of thick, elongated flakes.^85^, ^86^ This and subsequent cases of backing likely represent a more expedient, “cost‐effective” alternative to hafting. This simple edge modification quickly renders multiple flakes forms more ergonomic for use in “domestic” activities.^50^ Backing only appears as a systematic edge modification in the case of composite tools for hunting activities.^200^, ^201^, ^202^

With the development of new hunting strategies allowed by projectile technology, backing presents a series advantages: (1) it increases the robustness of both the microlith inserted and the haft, by removing the cutting potential of then edge; and (2) it allows pieces to be better fixed in the haft by creating a wider and rougher surface that increases the bond of the adhesive,^203^, ^204^ but see Pargeter et al.^205^ for a review. The progressive refinement of these increasingly correlated technologies eventually leads to the emergence of more standardized tools, which become characteristic elements of from late MSA in South and East Africa and from the Early Upper Paleolithic in Europe and likely included a cultural and social importance.^206^, ^207^, ^208^

## 6 FINAL REMARKS

Our review of the development of backing conception and the emergence of prepared backed tools sheds new light on Paleolithic cultural trajectories and innovations. Prepared and modified backs appeared in a mosaic fashion in different geographical regions and at different times across the Lower and, especially, the Middle Paleolithic. Backing seems to have emerged independently multiple times during Pleistocene human evolution, as highlighted by the diversity of tools and ecological and chrono‐cultural contexts. Current archeological data indicate retouched backed tools to have emerged in the late Middle Pleistocene at least in three different areas:

- the Levant, in the Amudian techno‐complex, between MIS 11‐9 and 7‐6, where typical backed knives were manufactured on elongated/laminar blanks, possibly by *H. heidelbergensis*/archaic *H. sapiens* populations in an environment characterized by a mosaic of open environment and woodland.^85^, ^97^
- Central Africa, in the debated Lupemban techno‐complex, between MIS 9‐7, in the form of small backed tools and tranchets in a rainforest environment.^89^, ^92^
- Possibly southern Europe in MIS 6, in the still only partially published San Francesco assemblage, which includes backed blades and truncations probably made by Neanderthals before the last interglacial.^102^, ^103^

The first evidence of backing thus includes highly diverse tool types in markedly different environments, accompanied at times by evidence for hafting. Technologically, two out of these three initial occurrences concern laminar assemblages, where naturally backed blades and flakes are well represented, while the latter (the Lupemban) is also associated with core‐axes, points, and blades.

Did this process of invention involve the slow acquisition and subsequent accumulation of the necessary techniques? Or, on the contrary, did innovations continuously appear and disappear multiple times before becoming a fixed element of hominin technology? The necessary techniques for “backing” stone tools are relatively simple, forming part of hominin technical repertoires from at least the late Lower Paleolithic and includes the use of bone retouchers.^209^, ^210^ With backed tools, unlike modifications to the working edges of stone tools, which are designed to improve performance, the retouching of backed tools concerns the passive part of the tool and is designed to improve its ergonomic aspect. This is conceptually similar to the bifacial shaping of handaxes and cleavers, which are often worked on the base for a better manipulation. Available data seems to point to a punctuated appearance of backing during the Middle Pleistocene and among multiple hominin species. This innovation primarily took the form of both improving the ergonomic proprieties of pre‐existing tools designs (i.e., natural or retouched backed flakes) and the emergence of new, more efficient tool types. This readjustment of the hominin tool kit is likely linked to increasing prehensive efficiency of backed tools and rendering them adaptable to diverse types of manipulation, from hand‐held to hafted tools.^27^, ^50^

The available data also suggests that the emergence of backing and hafting were not directly related. Instead, the invention and application of backing possibly emerged as a cost‐effective alternative to hafting. A chronological gap in the appearance of these two practices is usually present in most of the analyzed regions (Figure 7). Moreover, when the two techniques are simultaneously present in the same regions, they usually concern different kinds of blanks and stone tool kits. In any case, both hafting and backing are increasingly frequent from the late Middle Pleistocene in many parts of Africa, the Levant, and Europe, possibly linked to the same long and punctuated “revolution” marked by the emergence of Neanderthal and *H. sapiens* lineages, progressively under the influence of selective cultural pressures.^44^, ^95^, ^181^

If, as appears to be the case, an independent invention occurred in at least in three macro‐regions, these distinct technological traditions may have been transmitted to later generations. For example, in central Africa, post‐Lupemban assemblages include Mumbwa Caves (central Zambia), that produced several large segments (mostly crescent shaped) made on quartz from the last interglacial (130–116 ky).^211^ Similarities with the undated late MSA Polungu complex in Kalambo Falls have been noted^89^ as both assemblages are characterized by points, burins, blades, and curved or trapezoidal backed implements on flakes and blades obtained from very diverse core reduction methods. On the other hand, several late MSA assemblages emerging between MIS 4 and MIS 3 in many parts of southern and eastern Africa seem to belong to another tradition. Besides the chronological gap, the microlithic assemblages from Pinnacle Point (71.1 ± 2.3 ka),^212^ the Howiesons Poort complex (65–59 ka),^48^, ^200^, ^206^ and sites of the MSA/LSA transition in eastern Africa^213^, ^214^, ^215^ record coherent, small backed tools and segments linked to a blade/bladelet production. The proliferation of recurrent and standardized backed tools in these complexes has led them to interpreted as evidence of modern behavior, with a potential symbolic value reflected in the use of high‐quality, exotic raw materials.^48^, ^207^, ^216^

In Eurasia, the “tradition” of tool backing potentially present in southern Europe during MIS 6 and continues in laminar assemblages in the northern part of central‐western Europe (between the Rhine Valley, Belgium, and northern France) in MIS 5 (Figure 8). Here, the production of bladelets and points with backed retouch is a consistent feature of assemblages from a handful of sites. Re‐inventions also took place in the late Middle Paleolithic (early MIS 3), as evidence by the abundance and morpho‐typological consistency of backing in several Mousterian techno‐complexes, especially among discoidal and laminar assemblages in western and southern Europe^27^, ^47^, ^50^, ^119^, ^121^ (Figure 8).

In the Levant and North Africa, on the other hand, this technique seems to disappear after the Amudian and only reappear in the Early or Initial Upper Paleolithic industries. This discontinuity could also be related to the widespread hafting of Levallois blanks^186^, ^192^, ^217^ due to Levallois blanks possessing the morpho‐technical features which permit easier and safer hafting without backing.

This scenario would imply mechanisms of re‐invention or punctuated application of techniques from a pool of relatively complex technical know‐how which incorporated both backing and hafting. In the case of backed tools, this is a consequence of two factors: (1) it is a conceptually simple process; and (2) it allows immediate functional and ergonomic benefits.^50^ The re‐invention hypothesis is also supported by a general lack of standardization in these early contexts. Innovations are related to different functions and changes in tool prehension, suggested by the diversification of shapes, uses and types of manipulation. Moreover, these tools and related configurations are useful for both manual prehension and precision tasks as well as hafting. This variability seems to be particularly well expressed in discoidal contexts, while backed tools on elongated blanks show a higher degree of standardization.

The sporadic presence of backed tools in Lower and Middle Paleolithic assemblages may be linked by cultural and technological traditions under the influence of multiple elements, including ecological, demographic, and social factors.^17^, ^218^ Among these factors, is the appearance of laterality in human evolution, to which backing is possibly related. Precision and specialized activities necessarily involve a preferred laterality, which is related to higher specialization in tool use and precise/effective grasping.^219^, ^220^ Tool standardization involving pre‐configured morphologies may also be related to laterality. For instance, a preferred lateralization in the manufacturing of *keilmesser* or laminar‐backed knives is present in some Middle Paleolithic assemblages.^27^, ^121^, ^221^ The choice of which edge to back (in relation to the asymmetry in cross‐section and the main kinematic axis) strongly affects the preferred laterality in the use of the tool.

Despite the apparent “modernity” of such behaviors, a generally different conception of backed tools emerges within the European “transitional” techno‐complexes (Châtelperronian, Uluzzian), the Howiesons Poort and other MSA complexes and the African, Asian, and Australian microlithic assemblages, possibly appearing as a convergence of factors.^204^, ^222^ Here, a more pronounced tool standardization built into regularized blank production manufacturing is probably regulated by a conceptual scheme related to a precise function, specifically hafted projectile points, implying an abrupt change in hunting strategies.^200^, ^201^, ^206^, ^223^, ^224^ In these techno‐complexes, standardized blanks of particular sizes are produced with the clear intention of being backed, marking a clear rupture with almost all most of the previous contexts. This is in stark contrast to backing in the Middle Paleolithic, which appears more of an ‘after‐thought’ to blank production. Moreover, the punctuated, discontinuous appearance of backing in the Middle Paleolithic suggests it is more related to contextual contingencies of tool use (i.e., convergence) rather than distinct cultural traditions and that connecting techno‐complexes on either side of the Middle to Upper Paleolithic transition is, perhaps, overly simplistic.

The Late Paleolithic traditions in backing manufacturing are also easier to trace: the implements are characterized by a larger circulation network, potentially directly related to their function as projectiles. In these cases, tools could also serve as a means to signal social information about group identity and intercultural ties,^17^, ^89^, ^205^, ^206^ once again emphasizing the close relationships between technology, ecology and demography in Pleistocene hunter‐gatherer societies.

## 7 CONCLUSION

Here, we have attempted a critical review of the emergence of the backing concept, tracing its diffusion through the Lower and Middle Paleolithic of Africa and western Eurasia. To understand its long‐term evolutionary underpinnings, we investigated this aspect of stone tool transformation from the use of unmodified, naturally‐shaped backs to the intentional modification or creation of prepared backed artifacts, namely what we strictly consider “backing.” This evolutionary trajectory reflects a gradual increase in technical complexity of backed tools, which is expressed through the punctuated accumulation of technical and ergonomic knowledge and characterized by the appearance and disappearance of cultural innovations, as is evident in the sporadic presence of backing in Lower and Middle Paleolithic contexts.

We suggest that the emergence of the backing of stone tools is directly tied to the physical apparatus of the hominin hand and the ergonomics of tool use, including manual grips and hafting, occasionally linking to other technical innovations. The motivation behind the emergence of this behavior is likely multiple:

1. Selective pressure linked to the increased use of precision grasps and a reduction in tool size, where backing is related to miniaturization permitting a more efficient and safer grasp.
2. A need to broaden the corpus of artifacts that can be manipulated through blunting, a simpler ergonomic solution compared to hafting. Still, it can improve performance of blanks where these are less suited to hafting arrangements, being an intentional technical choice other than “economically” driven.
3. The invention of composite tools which include the insertion of small retouched blades and bladelets into organic shafts as part of new hunting strategies in the form of projectile technologies.

The variety of explanatory models is confirmed by the highly variable technical solutions evident in the production and transformation of these artifact types, ranging from elongated backed knives to small segments, including pseudo‐Levallois points and core‐edge flakes. The concept of backing is therefore not uniform in its expression but varies according to different sets of technological systems and practices. The invention of backing during the Lower to Middle Paleolithic appears to be context‐specific, emerging on multiple occasions and potentially incorporating elements of the conservation and transmission of technical know‐how, especially in some late Mousterian assemblages of Western Europe. In this latter scenario, backing would appear as a shared technical solution of Neanderthal groups across Eurasia applied to coeval yet technologically different stone tool production systems.

## Supporting information

Supporting information.

Supporting information.

## Data Availability

Not applicable.

## References

[1] Clark G. 1969. World prehistory: a new synthesis. Cambridge: Cambridge University Press.

[2] Muller A et al. 2022. Stone toolmaking difficulty and the evolution of hominin technological skills. Sci Rep 12:5883. 35393496 10.1038/s41598-022-09914-2 PMC8989887

[3] Foley R , Lahr MM. 2003. On stony ground: Lithic technology, human evolution, and the emergence of culture. Evol Anthropol Issues News Rev 12:109–122.

[4] Ambrose SH. 2001. Paleolithic technology and human evolution. Science 291:1748–1753. 11249821 10.1126/science.1059487

[5] Muller A , Clarkson C. 2016. Identifying major transitions in the evolution of lithic cutting edge production rates. PLoS One 11:1–23. 10.1371/journal.pone.0167244 PMC5147885 27936135

[6] Charbonnier G. 1961. Entretiens avec Claude Lévi‐Strauss. Plon Julliard.

[7] Simondon G. 1958. *On the mode of existence of technical objects, trans. Ninian Mel lamphy*. Unpubl Univ West Ontario London, Ontario.

[8] Cavalli‐Sforza LL , Feldman MW. 1981. Cultural transmission and evolution: a quantitative approach. Princeton, New Jersey: Princeton University Press. 7300842

[9] Lombao D et al. 2017. Teaching to make stone tools: new experimental evidence supporting a technological hypothesis for the origins of language. Sci Rep 7:1–14. 29089534 10.1038/s41598-017-14322-y PMC5663762

[10] Morgan TJH et al. 2015. Experimental evidence for the co‐evolution of hominin tool‐making teaching and language. Nat Commun. 6:1–8. 10.1038/ncomms7029 PMC4338549 25585382

[11] Tehrani JJ , Riede F. 2008. Towards an archaeology of pedagogy: learning, teaching and the generation of material culture traditions. World Archaeol 40:316–331.

[12] Tomasello M. 2009. The cultural origins of human cognition. Cambridge, MA: Harvard University Press.

[13] McBrearty S , Tryon C. 2006. From acheulean to middle stone age in the kapthurin formation, Kenya. Boston, MA: Springer US. p 257–277. 10.1006/jhev.2001.0513 11795975

[14] Wilkins J , Chazan M. 2012. Blade production ∼500 thousand years ago at Kathu Pan 1, South Africa: support for a multiple origins hypothesis for early Middle Pleistocene blade technologies. J Archaeol Sci 39:1883–1900.

[15] Mcbrearty S , Brooks AS. 2000. The revolution that wasn't: a new interpretation of the origin of modern human behavior. J Hum Evol 39:453–563. 11102266 10.1006/jhev.2000.0435

[16] Mellars P , Stringer CB. 1989. The human revolution: behavioural and biological perspectives on the origins of modern humans. Edimburgh: Edimburgh University Press.

[17] Scerri EML , Will M. 2023. The revolution that still isn't: the origins of behavioral complexity in *Homo sapiens* . J Hum Evol. 179:103358. 37058868 10.1016/j.jhevol.2023.103358

[18] Conard NJ. 2015. Cultural evolution during the Middle and Late Pleistocene in Africa and Eurasia. In: Henke W , Tattersall I , editors. Handb. paleoanthropology. Berlin, Heidelberg: Springer Berlin Heidelberg. p 2465–2508.

[19] d'Errico F , Stringer CB. 2011. Evolution, revolution or saltation scenario for the emergence of modern cultures? Philos Trans R Soc B Biol Sci 366:1060–1069. 10.1098/rstb.2010.0340 PMC3049097 21357228

[20] Sigault F. 1991. *Les points de vue constitutifs d'une science des techniques, essai de tableau comparatif*. Constr. une Sci. des Tech. Limonest: l'interdisciplinaire.

[21] Childe VG. 1956. Society and knowledge. London: Routledge.

[22] Migliano AB et al. 2020. Hunter‐gatherer multilevel sociality accelerates cumulative cultural evolution. Sci Adv. 6(9):eaax5913. 32158935 10.1126/sciadv.aax5913 PMC7048420

[23] Henrich J. 2001. Cultural transmission and the diffusion of innovations: adoption dynamics indicate that biased cultural transmission is the predominate force in behavioral change. Am Anthropol 103:992–1013.

[24] Shennan S. 2001. Demography and cultural innovation: a model and its implications for the emergence of modern human culture. Cambridge Archaeol J 11:5–16.

[25] Migliano AB , Vinicius L. 2022. The origins of human cumulative culture: from the foraging niche to collective intelligence. Philos Trans R Soc B Biol Sci 377:1843. 10.1098/rstb.2020.0317 PMC8666907 34894737

[26] Powell A et al. 2009. Late pleistocene demography and the appearance of modern human behavior. Science 324:1298–1301. 19498164 10.1126/science.1170165

[27] Delpiano D. 2021. *I coltelli degli ultimi Neandertal. Strategie tecnologiche e comportamentali alla fine del Paleolitico Medio*. British Archaeological Reports International Series.

[28] Bordes F. 1961. Typologie du Paléolithique inférieur et moyen. Bordeaux: Institut de Préhistoire de l'Université de Bordeaux I.

[29] Inizan M‐L et al. 1999. Préhistoire de la Pierre Taillée. Tome 5. Technology and Terminology of Knapped Stone.

[30] Turq A. 2000. *Paléolithique inférieur et moyen entre Dordogne et lot*. Société des Amis du Musée National de Préhistoire et de la Recherche Archéologique.

[31] De Sonneville‐Bordes D , Perrot J. 1956. Lexique typologique du Paléolithiquesupérieur. Bullettin la Société préhistorique française. 53:547–559.

[32] Barham L. 2013. From hand to handle: the first industrial revolution. Oxford: Oxford University Press.

[33] Bordes F. 1961. Mousterian cultures in France. Science 134:803–810. 17817388 10.1126/science.134.3482.803

[34] Debénath A , Dibble HL 1994. Handbook of Paleolithic typology vol. 1: the Lower and Middle Paleolithic of Europe. Philadelphia: University Museum, University of Pennsylvania.

[35] Peresani M. 2003. *Discoid lithic technology: advances and implications*. Archaeopre. British Archaeological Reports International Series.

[36] Jöris O. 2006. Bifacially backed knives (Keilmesser) in the Central European Middle Palaeolithic. In: Goren‐Inbar N , Sharon G , editors. Axe age, acheulian toolmak. from quarr. to discard. London: Equinox.

[37] Perera N et al. 2011. People of the ancient rainforest: Late Pleistocene foragers at the Batadomba‐lena rockshelter, Sri Lanka. J Hum Evol 61:254–269. 21777951 10.1016/j.jhevol.2011.04.001

[38] Pargeter J. 2016. Lithic miniaturization in Late Pleistocene southern Africa. J Archaeol Sci Rep 10:221–236.

[39] Slimak L. 2008. Sur un point de vue heuristique concernant la production et la transformation de support au Paléolithique moyen. Gall Préhistoire 50:1.22.

[40] Pargeter J , Shea JJ. 2019. Going big versus going small: lithic miniaturization in hominin lithic technology. Evol Anthropol 28:72–85. 30924224 10.1002/evan.21775

[41] Shea JJ , Sisk ML. 2010. Complex projectile technology and *Homo sapiens* dispersal into Western Eurasia. PaleoAnthropology. 2010:100–122.

[42] Key A , Lycett S. 2023. The ergonomics of stone tool use and production. In: Wynn T, Overmann KA, Coolidge F, editors. Oxford Handbook of Cognitive Archaeology . 1–25. Oxford University Press.

[43] Key A et al. 2021. Why invent the handle? Electromyography (EMG) and efficiency of use data investigating the prehistoric origin and selection of hafted stone knives. Archaeol Anthropol Sci 13:162.

[44] Mika A et al. 2023. Hafted technologies likely reduced stone tool‐related selective pressures acting on the hominin hand. Sci Rep Nat. 13:15582. 10.1038/s41598-023-42096-z PMC10511494 37730739

[45] Lepot M. 1993. *Approche techno‐fonctionnelle de l'outillage lithique moustérien: essai de classification des parties actives en termes d'efficacité technique*. Thése de doctorat, Université de Paris X‐ Nanterre.

[46] Boëda E. 2013. *Techno‐logique & technologie. Une Paléo‐histoire des objets lithiques tranchants*. Paris: @rchéo‐éditions.com.

[47] Ruebens K et al. 2015. On the local Mousterian origin of the Châtelperronian: integrating typo‐technological, chronostratigraphic and contextual data. J Hum Evol. 86:55–91. 26277304 10.1016/j.jhevol.2015.06.011

[48] Archer W. 2021. Carrying capacity, population density and the later Pleistocene expression of backed artefact manufacturing traditions in Africa. Philos Trans R Soc B Biol Sci 376:20190716. 10.1098/rstb.2019.0716 PMC7741103 33250028

[49] Leplongeon A et al. 2020. Backed pieces and their variability in the later stone age of the horn of Africa. Afr Arch Rev Afr Archaeol Rev 37:437–468.

[50] Delpiano D et al. 2019. Innovative neanderthals: results from an integrated analytical approach applied to backed stone tools. J Archaeol Sci 110:105011.

[51] Delagnes A et al. 2023. Long‐term behavioral adaptation of Oldowan toolmakers to resource‐constrained environments at 2.3 Ma in the Lower Omo Valley (Ethiopia). Sci Rep Nat 13:14350 10.1038/s41598-023-40793-3 PMC10474039 37658122

[52] de la Torre I , Mora R. 2018. Technological behaviour in the early Acheulean of EF‐HR (Olduvai Gorge, Tanzania). J Hum Evol. 120:329–377. 29706232 10.1016/j.jhevol.2018.01.003

[53] Caruana M V. et al. 2023. A techno‐functional analysis of acheulean backed knives from Wonderboom, South Africa. J F Archaeol 48:198–209.

[54] Key AJM et al. 2016. Looking at handaxes from another angle: assessing the ergonomic and functional importance of edge form in Acheulean bifaces. J Anthropol Archaeol. 44:43–55.

[55] Key A et al. 2018. Hand grip diversity and frequency during the use of Lower Palaeolithic stone cutting‐tools. J Hum Evol. 125:137–158. 30322659 10.1016/j.jhevol.2018.08.006

[56] Kleindienst M. 1962. Components of the East African Acheulian assemblage: an analytic approach. Actes du IVème Congrès Panafricain 40:81–99.

[57] Connet N et al. 2020. A 400,000 years old milestone of the Acheulian technocomplex in Central‐Western France at Londigny (Charente). J Archaeol Sci Rep 30:102225.

[58] Caruana MV et al. 2014. Quantifying traces of tool use: a novel morphometric analysis of damage patterns on percussive tools. PLoS One 9(11):e113856. 25415303 10.1371/journal.pone.0113856 PMC4240665

[59] Shea JJ. 2008. The Middle Stone Age archaeology of the Lower Omo Valley Kibish Formation: excavations, lithic assemblages, and inferred patterns of early *Homo sapiens* behavior. J Hum Evol 55:448–485. 18691735 10.1016/j.jhevol.2008.05.014

[60] Wurz S. 2002. Variability in the Middle Stone Age Lithic Sequence, 115,000–60,000 Years Ago at Klasies River, South Africa. J Archaeol Sci 29:1001–1015.

[61] Wadley L , Harper P. 1989. Rose cottage cave revisited: Malan's middle stone age collection. South African Archaeol Bull 44:23.

[62] Bourguignon L. 2001. Apports de l'expérimentation et de l'analyse techno‐morpho‐fonctionelle à la reconnaissance du processus d'aménagement de la retouche Quina. In: Bourguignon L et al., editors. Préhistoire approach. expérimentale. Montagnac, Éditions Monique Mergoil. p 35–66.

[63] Bourguignon L. 1996. La conception de débitage Quina. Quat Nov VI:149–166.

[64] Delpiano D et al. 2022. Flexibility within Quina lithic production systems and tool‐use in Northern Italy: implications on Neanderthal behavior and ecology during early MIS 4. Archaeol Anthropol Sci 14:219.

[65] Hiscock P et al. 2009. Quina procurement and tool production. In: Adams B , Blades BS , editors. Lithic mater paleolit soc. Chichester: Wiley‐Blackwell. p 232–246.

[66] Turq A. 1992. Raw material and technological studies of the Quina Mousterian. In: Dibble HL , Mellars P , editors. Middle palaeolithic adapt behav. Var. Philadelphia: University of Pennsylvania.

[67] Forestier H. 2009. Le Clactonien: mise en application d'une nouvelle méthode de débitage s'inscrivant dans la variabilité des systèmes de production lithique du Paléolithique ancien. Paléo 5:53–82.

[68] Geneste J , Plisson H. 1996. Production et utilisation de l'outillage lithique dans le Moustérien du sudouest de la France: les Tares à Sourzac, Vallé de l'Isle, Dordogne. Reduction Processes («Chaînes Opératoires») for the European Mousterian. Quat Nov VI :343–367.

[69] Faivre J et al. 2009. La fracturation en split, une technique de production dans l'industrie lithique des Tares (Sourzac, Dordogne). Paléo :123–132.

[70] Boëda E. 1993. Le débitage discoïde et le débitage Levallois récurrent centripète Bullettin la Société Préhistorique Française 90(6):392‐2010404.

[71] Delpiano D et al. 2021. Techno‐functional implication on the production of discoid and levallois backed implements techno‐functional implication on the production of discoid and levallois backed implements. Lithic Technol. 46(3):171–191.

[72] Gravina B , Discamps E. 2015. MTA‐B or not to be? Recycled bifaces and shifting hunting strategies at Le Moustier and their implication for the late Middle Palaeolithic in southwestern France. J Hum Evol 84:83–98. 25976251 10.1016/j.jhevol.2015.04.005

[73] Thomas M , Gravina B. 2019. Analyse techno‐économique d'un assemblage Discoïde du Moustérien récent de l'abri inférieur du Moustier (Dordogne, France). Paléo. 30(1):300–317.

[74] Sandgathe DM. 2005. *An analysis of the levallois reduction strategy using a design theory framework*. PhD Thesis, Simon Fraser University.

[75] Boëda E. 1994. Le concept Levallois: variabilité des méthodes. Paris: CNRS.

[76] Bustos‐Pérez G et al. 2023. What lies in between: Levallois, discoid and intermediate methods. J Lithic Stud 10(2):32.

[77] Marks AE et al. 2002. Le gisement pléistocène moyen de Galeria Pesada (Estrémadure, Portugal); premiers résultats. Paléo 14:77–100.

[78] Solecki RL , Solecki RS. 2004. Bifaces and the Acheulian industries of Yabroud Shelter I, Syria. In: Toussaint M et al., editors. Gen sess posters sect 4 hum orig low palaeolithic acts XIVth UISPP congr. liège 2001. Oxford: British Archaeological Reports International Series. p 37–39.

[79] Frick JA. 2020. Reflections on the term Micoquian in Western and Central Europe. Change in criteria, changed deductions, change in meaning, and its significance for current research. Archaeol Anthropol Sci Archaeol Anthropol Sci. 12, 38.

[80] Rots V. 2009. The functional analysis of the Mousterian and Micoquian assemblages of Sesselfelsgrotte, Germany: aspects of tool use and hafting in the European Late. Quartär 56:37–66.

[81] Delpiano D , Uthmeier T. 2020. Techno‐functional and 3D shape analysis applied for investigating the variability of backed tools in the Late Middle Paleolithic of Central Europe. PLoS One. 15(8):e0236548. 32813722 10.1371/journal.pone.0236548 PMC7446931

[82] Boëda E. 1995. Caractéristiques techniques des chaînes opératoires lithiques des niveaux micoquiens de Külna (Tchécoslovaquie). Paléo Suppl. 1:57–72.

[83] Iovita R. 2014. The role of edge angle maintenance in explaining technological variation in the production of Late Middle Paleolithic bifacial and unifacial tools. Quat Int. 350:105–115.

[84] Migal W , Urbanowski M. 2006. Pradnik knives reuse. Experimental approach. In: Wisniewski A et al., editors. Stone tech technol. Wroclaw: Uniwersytet Wrocławski. p 73–89.

[85] Barkai R et al. 2009. A blade for all seasons? Making and using Amudian blades at Qesem Cave, Israel. Hum Evol 24:57–75.

[86] Barkai R et al. 2006. Middle Pleistocene blade production in the Levant: an Amudian assemblage from Qesem Cave, Israel. Eurasian Prehistory 3:39–74.

[87] Shimelmitz R et al. 2011. Systematic blade production at late Lower Paleolithic (400—200 kyr) Qesem Cave. J Hum Evol 61:458–479. 21813161 10.1016/j.jhevol.2011.06.003

[88] Meignen L. 2000. Early Middle Palaeolithic blade technology in Southwestern Asia. Acta Anthropol Sin 19:158–168.

[89] Barham L. 2002. Backed tools in Middle Pleistocene central Africa and their evolutionary significance. J Hum Evol 43:585–603. 12457850 10.1006/jhev.2002.0597

[90] Clark JD. 1959. The prehistory of Southern Africa. Harmondsworth: Penguin Book.

[91] Taylor N. 2016. *Across rainforests and woodlands: a systematic reappraisal of the Lupemban Middle Stone Age in Central Africa*. In: Jones SC, Stewart BA, editors. Africa from MIS 6‐2. Population Dynamics and Paleoenvironments. p 273–299.

[92] Clark JD. 2001. Variability in primary and secondary technologies of the Later Acheulian in Africa. In: Milliken S , Cook J , editors. A very remote period indeed pap paleolit present to derek roe. Oxford: Oxbow Books. p 1–18.

[93] Clark JD et al. 1947. New studies on Rhodesian Man. J R Anthropol Inst 77:7–32.

[94] Grün R et al. 2020. Dating the skull from Broken Hill, Zambia, and its position in human evolution. Nature 580:372–375. 32296179 10.1038/s41586-020-2165-4

[95] Rots V et al. 2011. Aspects of tool production, use, and hafting in Palaeolithic assemblages from Northeast Africa. J Hum Evol. 60:637–664. 21392816 10.1016/j.jhevol.2011.01.001

[96] Van Peer P et al. 2004. A story of colourful diggers and grinders: the Sangoan and Lupemban at site 8‐B‐11, Sai Island, Northern Sudan. Before Farming 1:1–28.

[97] Mercier N et al. 2013. New datings of Amudian layers at Qesem Cave (Israel): results of TL applied to burnt flints and ESR/U‐series to teeth. J Archaeol Sci. 40:3011–3020.

[98] Lemorini C et al. 2006. Use‐wear analysis of an Amudian laminar assemblage from the Acheuleo‐Yabrudian of Qesem Cave, Israel. J Archaeol Sci 33:921–934.

[99] Copeland L. 1983. The Paleolithic stone industries. In: Roe D , editor. Adlun stone age excav D.A.E. Garrod Lebanon 1958‐1963. Oxford: BAR International Series.

[100] Jelinek A. 1975. A preliminary report on some Lower and Middle Paleolithic Industries from the Tabun Cave, (Mount Carmel), Israel. In: Wendorf R , Marks AE , editors. Probl prehistory North Africa levant. Dallas: SMU Press. p 297–315.

[101] Groman‐Yaroslavski I et al. 2021. Complexity and sophistication of Early Middle Paleolithic flint tools revealed through use‐wear analysis of tools from Misliya Cave, Mount Carmel, Israel. J Hum Evol. 154:102955. 33831631 10.1016/j.jhevol.2021.102955

[102] Tavoso A. 1988. L'outillage du gisement de San Francesco a San Remo (Ligurie, Italie): nouvel examen. In: Kozlowski JK , editor. l'Homme Néandertal 8 La Mutat. Liège: ERAUL 36. p 193–210.

[103] Pirouelle F. 2006. *Contribution méthodologique à la datation, par les méthodes Uranium‐Thorium (U‐TH) et résonancede spin électronique (ESR), de sites moustériens de Ligurie, de France et de Belgique*. Muséum d'Histoire Naturelle, Paris.

[104] Klostermann J , Thissen J. 1995. Die stratigraphische Stellung des Lößprofils von Mönchengladbach‐Rheindahlen (Niederrhein). E&G Quat Sci J 45:42–58.

[105] Conard NJ , Adler DS. 1997. Lithic reduction and hominid behavior in the Middle Paleolithic of the Rhineland. J Anthropol Res 53:147–175.

[106] Conard NJ. 1997. Middle Palaeolithic subsistence in the central Rhine valley. Anthropozoologica 25–26:329–336.

[107] Cyrek K et al. 2014. Middle palaeolithic cultural levels from Middle and late Pleistocene sediments of Biśnik Cave, Poland. Quat Int. 326‐327:20–63.

[108] Vande Walle H. 2003. La production des outils au Paléolithique moyen: comparaison diachronique des occupations de Riencourt‐lès‐Bapaume (Pas‐de‐Calais, France). Paléo. 15:169–194.

[109] Bietti A , Negrino F. 2007. The transition between Mousterian and Aurignacian industries in continental Italy: a status report. In: Riel‐Salvatore J , Clark GA , editors. Transitions Gt. Small New Approaches to Study Early Up. Paleolit. ‘Transitional’ Ind. West Eurasia. Oxford: Archaeopress. p 41–59.

[110] Tuffreau A et al. 1994. Le gisement paléolithique moyen de Seclin (Nord). Bull la Société Préhistorique Française 91:23–46.

[111] Conard NJ , Prindiville TJ. 2000. Middle Palaeolithic hunting economies in the Rhineland. Int J Osteoarchaeol 10:286–309.

[112] Boëda E. 1988. Le concept laminaire: rupture et filiation avec le concept Levallois. Liège 8:41–59.

[113] Cahen D , Haesaerts P. 1984. *Peuples chasseurs de la Belgique Préhistorique dans leur cadre naturel*. Bruxelles: Patrimoine de l'Institut royal des Sciences naturelles de Belgique.

[114] Sytnyk O et al. 2010. The Dniesterian Mousterian from the Velykyi Glybochok site related to palaeoenvironmental changes. Quat Int 220:31–46.

[115] Bordes F. 1954. Les gisements du Pech de l'Azé (Dordogne). I. Le Moustérien de tradition acheuléenne. (avec une note Paléontologique de J. Bouchud). Anthropologie 58:401–432.

[116] Mellars P. 1996. The neanderthal legacy. An archaeological perspective from Western Europe. Princeton University Press.

[117] Faivre J et al. 2014. *The contribution of lithic production systems to the interpretation of Mousterian industrial variability in south‐western France: the example of Combe‐Grenal (Dordogne, France)*. Quat Int Elsevier Ltd.

[118] Faivre JP et al. 2017. Late Middle Palaeolithic lithic technocomplexes (MIS 5–3) in the northeastern Aquitaine Basin: advances and challenges. Quat Int. 433:116–131.

[119] Gravina B. 2016. *La fin du Paléolithique moyen en Poitou‐Charentes et Périgord: Considérations à partir de l'étude taphonomique et techno‐économique des sites du Moustier (niveaux G à K) et La Roche‐à‐Pierrot, Saint Césaire (niveau EJOP supérieur)*. Thése de doctorat, Université de Bordeaux.

[120] Roussel M , Soressi M. 2010. La Grande Roche de la Plematrie a Quinçay (Charente‐Maritime): Nouvelles donnes sur l'industrie lithique du Chatelperronien. In: Buisson‐Catil JP , editor. Préhistoire entre Vienne Charente Hommes Sociétés du Paléolithique. Chauvigny p 203–220.

[121] Soressi M. 2002. *Le Moustérien de tradition acheuléenne du sud‐ouest de la France*. Doctoral Thesis, Université de Bordeaux.

[122] Thiébaut C. 2005. *Le Moustérien à denticulés: Variabilité ou diversité techno‐économique?* Tome1. Problématique et méthodologie.

[123] Turq A et al. 2011. Les fouilles récentes du Pech de l'Azé IV (Dordogne). Gall Préhistoire 53:1–58.

[124] Locht J , Swinnen C. 1994. Le débitage discoïde du gisement de Beauvais (Oise): aspects de la chaîne opératoire au travers de quelques remontages. Paléo 6:89–104.

[125] Slimak L et al. 2022. Modern human incursion into Neanderthal territories 54,000 years ago at Mandrin, France. Sci Adv 8:1–17. 10.1126/sciadv.abj9496 PMC8827661 35138885

[126] Bodu P et al. 2014. An open‐air site from the recent Middle Palaeolithic in the Paris Basin (France): Les Bossats at Ormesson (Seine‐et‐Marne). Quat Int Elsevier Ltd and INQUA. 331:39–59.

[127] Thiébaut C et al. 2009. Les derniéres occupations moustériennes de Saint‐Césaire (Charente‐Maritime, France). Bull la Société Préhistorique Française 106:691–714.

[128] Duran JP , Tavoso A. 2005. Les industries moustériennes de la Rouquette (Puycelci, Tarn, France). Anthropologie 109:755–783.

[129] Ulrix‐closset M. 1975. Le Paléolithique moyen dans le bassin mosan en Belgique. Wetteren: Univers.

[130] Delpiano D et al. 2018. Assessing Neanderthal land use and lithic raw material management in Discoid technology. *J Anthropol Sci* 96:1–22. 10.4436/JASS.96006 30153108

[131] Bourguignon L , Turq A. 2003. Une chaine opératoire de débitage discoide sur eclat du Mousterien à denticules Aquitain: les exemples des ChampBossuet et de Combe‐Grenal c.14. In: Peresani M , editor. *Discoid lithic technol adv implic*. British Archaeological Reports International Series.

[132] Gravina B. 2014. *La fin du Paléolithique moyen en Poitou‐Charentes et Périgord: Considérations à partir de l'étude taphonomique et techno‐économique des sites du Moustier (niveaux G à K) et La Roche‐à‐Pierrot, Saint Césaire (niveau EJOP supérieur)*. Université de Bordeaux.

[133] Jelinek A. 2013. Neanderthal lithic industries at La Quina. Tucson: The University of Arizona Press.

[134] Vacca V. 2022. *Variabilité d'un outillage néandertalien: quelles implications sur l'utilisation et les gestes? Approche morphométrique, tracéologique et expérimentale appliquée à des pointes pseudo‐Levallois issues du site du Moustier (Dordogne)*. Université Toulouse Jean Jaurès.

[135] Locht J. 2003. L'industrie lithique du gisement de Beauvais (Oise, France): Objectifs et variabilité du débitage discoïde. In: Peresani M , editor. *Discoid lithic technol adv implic*. British Archaeological Reports International Series.

[136] Metz L. 2023. Post‐Néronien I. Analyse fonctionnelle des pointes pseudo‐Levallois de la couche D. In: Slimak L et al., editors. Mandrin Des derniers néandertaliens aux premiers hommes Mod. en Fr. méditerranéenne . Aix‐en‐Provence: Artisanats & Territoires. p 582–595.

[137] Bourguignon L et al. 2002. L'habitat moustérien de « La Folie » (Poitiers, Vienne): synthèse des premiers résultats. Paléo 14:29‐48.

[138] Gonzalez Echegaray J , Freeman LG. 1971. *Cueva Morin: Excavaciones 1966‐68*. Santander: Publicaciones del Patronato de las Cuevas Prehistoricas de la Provincia de Santander.

[139] Di Modica K et al. 2016. The Middle Palaeolithic from Belgium: chronostratigraphy, territorial management and culture on a mosaic of contrasting environments. Quat Int 411:77–106.

[140] Degros J et al. 1982. Un gisement du Paléolithique moyen à La Madeleine‐sur‐Loing (Seine‐et‐Marne). Bull la Société Préhistorique Française 79:330–340.

[141] Boëda E et al. 1996. Barbas III. Industries du Paléolithique Moyen récent et du Paléolithique Supérieur ancien. In: Carbonell E , Vaquero M , editors. Last Neanderthals first anat mod humans . p 147–156.

[142] Key AJM. 2016. Integrating mechanical and ergonomic research within functional and morphological analyses of lithic cutting technology: key principles and future experimental directions. Ethnoarchaeology 8:69–89.

[143] Tocheri MW et al. 2008. The evolutionary history of the hominin hand since the last common ancestor of Pan and Homo. J Anat 212:544–562. 18380869 10.1111/j.1469-7580.2008.00865.x PMC2409097

[144] Kivell TL. 2015. Evidence in hand: recent discoveries and the early evolution of human manual manipulation. Philos Trans R Soc B Biol Sci 370:20150105. 10.1098/rstb.2015.0105 PMC4614723 26483538

[145] Kunze J et al. 2022. Entheseal patterns suggest habitual tool use in early hominins. Paleoanthropology 2022:195–210.

[146] Kilpatrick SJ. 2021. Human physiology and acheulean handaxe design. Doctoral Thesis. Department of Anthropology, University of Toronto.

[147] Feix T et al. 2009. A comprehensive grasp taxonomy. Robot Sci Syst Conf Work Underst Hum Hand Adv Robot Manip :2–3.

[148] Feix T et al. 2016. The GRASP taxonomy of human grasp types. IEEE Trans Hum Mach Syst 46:66–77.

[149] Fedato A et al. 2020. Hand grasping and finger flexion during Lower Paleolithic stone tool ergonomic exploration. Archaeol Anthropol Sci 12.

[150] Silva‐Gago M et al. 2019. A preliminary survey on hand grip and hand‐tool morphometrics in three different stone tools. J Archaeol Sci Rep. 23:567–573.

[151] Keeley LH , Toth N. 1981. Microwear polishes on early stone tools from Koobi Fora, Kenya. Nature. 293:464–465.

[152] Toth N. 1997. The artefact assemblages in the light of experimental studies. In: Isaac GL , editor. Koobi fora research project. Oxford: Clarendon Press. p 363‐401.

[153] Beyries S. 1993. Are we able to determine the function of the earliest palaeolithic tools? In: Berthelet A, Chavaillon J, editors. The Use of tools by human and non‐human primates. Oxford University Press. p 225–236.

[154] Sussman L. 1987. Résultats d'une étude des microtraces d'usure sur un échantillon d'artefacts d'Olduvai (Tanzanie). Anthropologie 91:375–380.

[155] Binneman J , Beaumont P. 1992. Use wear analysis of two Acheulean handaxes from Wonerwerk Cave, Northern Cape. South African F Archaeol. 1:92–97.

[156] Zupancich A et al. 2021. Biface use in the Lower Paleolithic Levant: first insights from late Acheulean Revadim and Jaljulia (Israel). J Archaeol Sci Rep 36:102877.

[157] Groman‐Yaroslavski I et al. 2022. Tool wielding and activities at the Middle Paleolithic site of Nesher Ramla, Israel: a use‐wear analysis of major tool types from unit III. Quat Int 624:67–79.

[158] Vallin L et al. 2006. L'outil idéal. Analyse du standard Levallois des sites moustériens d'Hermies (Nord de la France). Paléo 18:237–272.

[159] Rots V. 2011. Tool Use and Hafting in the Western European Middle Palaeolithic. Le Paléolithique moyen en Belgique Mélanges Marguerite Ulrix‐Closset 4:277–287.

[160] Beyries S , Boëda E. 1983. Etude technologique et traces d'utilisation des “éclats dèbordants” de Corbéhem (Pas‐de‐Calais). Bull la Soc Préhistorique Française 80:275–279.

[161] Arrighi S et al. 2009. Production and use in the lithic industry of the Mousterian in Santa Croce (Bisceglie, Italy). Hum Evol 24:91–106.

[162] Thiébaut C et al. 2014. Diversité des productions lithiques du Paléolithique moyen récent (OIS 4‐OIS 3): enquête sur le rôle des facteurs environnementaux, fonctionnels et culturels. In: Jaubert J et al., editors. *Transitions, ruptures Contin. en Préhistoire*. Bordeaux ‐ Les Eyzies p 281–296.

[163] Veil S et al. 1994. Ein mittelpalaolithischer Fundplatz aus der Weichsel‐Kaltzeit bei Lichtenberg. Landkreis Lüchow‐Dannenberg. Zwi‐ schenbericht über die archaologischen und geowissenschaftlichen Untersuchungen 1987‐1992. *Germania* 72:1–65.

[164] Claud É et al. 2012. Étude tracéologique de l'outillage moustérien de type Quina du bonebed de Chez‐Pinaud à Jonzac (Charente‐Maritime). Nouveaux éléments en faveur d'un site de boucherie et de traitement des peaux. Gall Préhistoire 54:3–32.

[165] Brenet M et al. 2017. The function and role of bifaces in the Late Middle Paleolithic of southwestern France: examples from the Charente and Dordogne to the Basque Country. Quat Int 428:151–169.

[166] Bourguignon L et al. 2004. Ramification des chaînes opératoires: une spécificité du Moustérien? Paléo 16:37–48.

[167] Anderson‐Gerfaud P. 1981. *Contribution méthodologique à l'analyse des microtraces d'utilisations sur les outils préhistoriques*. Thèse de 3ème cycle de l'Université de Bordeaux I.

[168] Carpentieri M et al. 2023. Brief interviews with hideous stone: a glimpse into the butchery site of Isernia La Pineta—a combined technological and use‐wear approach on the lithic tools to evaluate the function of a Lower Palaeolithic context. Archaeol Anthropol Sci. 15:93.

[169] Starkovich BM et al. 2020. Minimal tools, maximum meat: a pilot experiment to butcher an elephant foot and make elephant bone tools using lower paleolithic stone tool technology. Ethnoarchaeology. 12:118–147.

[170] Aureli D et al. 2015. Palaeoloxodon and human interaction: depositional setting, chronology and archaeology at the Middle Pleistocene Ficoncella site (Tarquinia, Italy). PLoS One 10:1–27. 10.1371/journal.pone.0124498 PMC4405345 25898322

[171] Lemorini C et al. 2023. Life around the elephant in space and time: an integrated approach to study the human‐elephant interactions at the Late Lower Paleolithic site of La Polledrara di Cecanibbio (Rome, Italy). J Archaeol Method Theory. 30:1233‐1281.

[172] Tourloukis V et al. 2018. Lithic artifacts and bone tools from the Lower Palaeolithic site Marathousa 1, Megalopolis, Greece: Preliminary results. Quat Int. 497:47–64.

[173] Abruzzese C et al. 2016. Assessment of the Acheulean in Southern Italy: new study on the Atella site (Basilicata, Italy). Quat Int. 393:158–168.

[174] Venditti F et al. 2019. Techno‐functional analysis of small recycled flakes from Late Acheulian Revadim (Israel) demonstrates a link between morphology and function. J Archaeol Sci Rep. 28:102039.

[175] Venditti F et al. 2022. Using microartifacts to infer Middle Pleistocene lifeways at Schöningen, Germany. Sci Rep Nat. 12:1–15. 10.1038/s41598-022-24769-3 PMC9755147 36522355

[176] Moyano IT et al. 2011. The archaic stone tool industry from Barranco León and Fuente Nueva 3, (Orce, Spain): Evidence of the earliest hominin presence in southern Europe. Quat Int. 243:80–91.

[177] Alperson‐Afil N , Goren‐Inbar N. 2016. Acheulian hafting: proximal modification of small flint flakes at Gesher Benot Ya'aqov, Israel. Quat Int 411:34–43.

[178] Niewoehner WA et al. 2003. Manual dexterity in Neanderthals. Nature 422:395. 10.1038/422395a 12660770

[179] Karakostis FA et al. 2018. Evidence for precision grasping in Neandertal daily activities. *Sci Adv* 4:1–12. 10.1126/sciadv.aat2369 PMC6157967 30263956

[180] Rots V. 2013. Insights into early Middle Palaeolithic tool use and hafting in Western Europe. The functional analysis of level IIa of the early Middle Palaeolithic site of Biache‐Saint‐Vaast (France). J Archaeol Sci 40:497–506.

[181] Mazza PPA et al. 2006. A new Palaeolithic discovery: tar‐hafted stone tools in a European Mid‐Pleistocene bone‐bearing bed. J Archaeol Sci 33:1310–1318.

[182] Pawlik AF , Thissen JP. 2011. Hafted armatures and multi‐component tool design at the Micoquian site. J Archaeol Sci. 38:1699–1708.

[183] Koller J et al. 2001. High‐tech in the middle palaeolithic: neandertal‐manufactured pitch identified. Eur J Archaeol 4:385–397.

[184] Degano I et al. 2019. Hafting of Middle Paleolithic tools in Latium (central Italy): new data from Fossellone and Sant'Agostino caves. PLoS One. 14(10):e0223714. 31220106 10.1371/journal.pone.0213473 PMC6586293

[185] Niekus MJLT et al. 2019. Middle paleolithic complex technology and a Neandertal tar‐backed tool from the Dutch North Sea. Proc Natl Acad Sci USA 116:22081–22087. 31636186 10.1073/pnas.1907828116 PMC6825292

[186] Boëda E et al. 2008. Middle Palaeolithic bitumen use at Umm el Tlel around 70 000 BP. Antiquity 82:853–861.

[187] Hauck TC et al. 2013. Molecular evidence of bitumen in the Mousterian lithic assemblage of Hummal (Central Syria). J Archaeol Sci. 40:3252–3262.

[188] Carciumaru R et al. 2012. New evidence of adhesive as hafting material on Middle and Upper Palaeolithic artefacts from Gura Cheii‐Râsnov Cave (Romania). J Archaeol Sci 39:1942–1950.

[189] Schmidt P et al. 2024. Ochre‐based compound adhesives at the Mousterian type‐site document complex cognition and high investment. Sci Adv 10:822. 10.1126/sciadv.adl0822 PMC10881035 38381827

[190] Rots V. 2010. Prehension and hafting traces on flint tools: a methodology. Universitaire Pers Leuven.

[191] Rots V. 2003. Towards an understanding of hafting: the macro‐ and microscopic evidence. Antiquity 77:805–815.

[192] Martin‐Viveros JI et al. 2023. Butchering knives and hafting at the Late Middle Paleolithic open‐air site of Nahal Mahanayeem Outlet (NMO), Israel. Sci Rep Nature. 13:1–18. 10.1038/s41598-022-27321-5 PMC9810700 36596848

[193] Rots V. 2015. *Hafting and site function in the European Middle Paleolithic*. In Conard N, Delagnes A, editors. Settlement Dynamics of the Middle Palaeolithic and the Middle Stone Age, Volume IV. 383‐411.

[194] Bonilauri S. 2010. *Les outils du Paléolithique moyen: une mémoire technique oubliée? Approche techno‐fonctionnelle appliquée à un assemblage lithique de conception Levallois provenant du site d'Umm el Tlel (Syrie centrale)*. Doctoral dissertation, Université Paris X‐ Nanterre.

[195] Rots V , Plisson H. 2014. Projectiles and the abuse of the use‐wear method in a search for impact. J Archaeol Sci. 48:154–165.

[196] Porraz G. 2002. Les pièces amincies de la Baume des Peyrards (Massif du Luberon, Vaucluse): analyse des procédés de réalisation. Prehistoires Mediteranées. 27‐28:10–11.

[197] Stordeur D. 1987. La main et l'outil: manches et emmanchements préhistoriques. Lyon: Maison de l'Orient Mediterranéen.

[198] Tryon CA et al. 2006. Levallois lithic technology from the Kapthurin formation, Kenya: Acheulian Origin and Middle Stone Age diversity. African Archaeol Rev 22:199–229.

[199] Tomasso S , Rots V. 2018. What is the use of shaping a tang? Tool use and hafting of tanged tools in the Aterian of Northern Africa. Archaeol Anthropol Sci. 10:1389–1417.

[200] Villa P et al. 2010. The Howiesons Poort and MSA III at Klasies River main site, Cave 1A. J Archaeol Sci. 37:630–655.

[201] Wurz S , Lombard M. 2007. 70 000‐year‐old geometric backed tools from the Howiesons Poort at Klasies River, South Africa: were they used for hunting?. South Afr Humanit 19:1–16.

[202] Lombard M , Phillipson L. 2010. Indications of bow and stone‐tipped arrow use 64 000 years ago in KwaZulu‐Natal, South Africa. Antiquity 84:635–648.

[203] Andrew MZ et al. 2014. An experimental study of hafting adhesives and the implications for compound tool technology. PLoS One 9(11):e112560. 25383871 10.1371/journal.pone.0112560 PMC4226580

[204] Clarkson C et al. 2018. *Small, sharp, and standardized: global convergence in Backed‐Microlith technology*.

[205] Pargeter J et al. 2022. Stone tool backing and adhesion in hunting weaponry: First results of an experimental program. J Archaeol Sci Reports 45:103639.

[206] Way AM et al. 2022. Howiesons Poort backed artifacts provide evidence for social connectivity across southern Africa during the Final Pleistocene. Sci Rep Nat. 12:1–12. 10.1038/s41598-022-12677-5 PMC9184481 35680943

[207] Ambrose SH. 2002. Small things remembered: origins of Early Microlithic Industries in Sub‐Saharan Africa. Archaeol Pap Am Anthropol Assoc 12:9–29.

[208] Peresani M et al. 2019. The Uluzzian in the north of Italy: insights around the new evidence at Riparo Broion. Archaeol Anthropol Sci 11:3503–3536.

[209] Rosell J et al. 2011. Bone as a technological raw material at the Gran Dolina site (Sierra de Atapuerca, Burgos, Spain). J Hum Evol 61:125–131. 21420719 10.1016/j.jhevol.2011.02.001

[210] Julien M‐A et al. 2015. Characterizing the Lower Paleolithic bone industry from Schöningen 12 II: a multi‐proxy study. J Hum Evol 89:264–286. 26651609 10.1016/j.jhevol.2015.10.006

[211] Barham L. 2000. The Middle Stone Age of Zambia, South Central Africa. Liverpool: Western Academic & Specialist Press.

[212] Brown KS et al. 2012. An early and enduring advanced technology originating 71,000 years ago in South Africa. Nature. 491:590–593. 23135405 10.1038/nature11660

[213] Tryon CA et al. 2018. Middle and later stone age chronology of Kisese II rockshelter (UNESCO World Heritage Kondoa Rock‐Art Sites), Tanzania. PLoS One 13:1–24. 10.1371/journal.pone.0192029 PMC5830042 29489827

[214] Shipton C et al. 2018. 78,000‐year‐old record of Middle and Later stone age innovation in an East African tropical forest. Nat Commun. 9:1–8. 29743572 10.1038/s41467-018-04057-3 PMC5943315

[215] Leplongeon A. 2014. Microliths in the Middle and Later Stone Age of Eastern Africa: new data from Porc‐Epic and Goda Buticha cave sites, Ethiopia. Quat Int. 343:100–116.

[216] Wurz S. 1999. The Howiesons poort backed artefacts from Klasies River: an argument for symbolic behaviour. South African Archaeol Bull 54:38.

[217] Boëda E et al. 1999. A Levallois point embedded in the vertebra of a wild ass (*Equus africanus*): hafting, projectiles and Mousterian hunting weapons. Antiquity 73:394–402.

[218] Kandel AW et al. 2016. Increasing behavioral flexibility? An integrative macro‐scale approach to understanding the Middle Stone Age of Southern Africa. J Archaeol Method Theory. 23:623–668.

[219] Wynn T. 1989. *The evolution of spatial competence*. Illinois Studies in Communication. Universoty of Illinois Press.

[220] Cashmore L et al. 2008. The evolution of handedness in humans and apes: a review and current issues. J Anthropol Sci 86:7–35. 19934467

[221] Jöris O , Uomini N. 2017. Evidence for Neanderthal hand‐preferences from the late Middle Palaeolithic site of Buhlen, Germany: insights into Neanderthal learning behaviour. In: Nishiaki Y , Jöris O , editors. Learn strateg dur palaeolithic. Tokyo: Springer.

[222] Lewis L. 2017. *Early microlithic technologies and behavioural variability in Southern Africa and South Asia*. BAR International Series.

[223] Lombard M , Pargeter J. 2008. Hunting with Howiesons Poort segments: pilot experimental study and the functional interpretation of archaeological tools. *J Archaeol Sci* 35:2523–2531.

[224] Pelegrin J , Soressi M. 2007. Le Chatelperronien et ses rapports avec le Moustérien. In: Vandermeersch B et al., editors. Les Néandertaliens Biol Cult Biol Cult. Paris: Comité des travaux historiques et scientifiques. p 283–296.

