# Supplementary material for: Back(s) to basics: The concept of backing in stone tool technologies for tracing hominins' technical innovations
Source: Evol Anthropol. 2024 Aug 7;33(6):e22045. doi: 10.1002/evan.22045 (PMC11624295; doi:10.1002/evan.22045)
Supplement: Supplementary file 2 — Supporting information. [file EVAN-33-e22045-s001.docx]

1. **Grotta di Fumane**

1.1 Stratigraphy

Fumane Cave is located about 350 meters above sea level, in the southern part of the Lessini Mountains, in the province of Verona. It opens to the south within Jurassic limestones (San Vigilio Oolites), in a narrow valley (Vajo di Manune) that is a right tributary of the main Vajo di Fumane, which directly connects it to the nearby Holocene plain (Fig. SI1.1). The archaeological deposit was initially disturbed in the lower levels near a road during its widening in 1964. The site was initially named Riparo Solinas (Solinas rockshelter) after its discoverers G. Solinas and F. Mezzena first investigated the stratigraphy, and because the inner cavity was then completely blocked by sediment. When research resumed in the 1980s, and then systematically carried out from 1988 under the joint direction of the University of Ferrara (Prof. A. Broglio) and the University of Milan (Prof. M. Cremaschi), three galleries were discovered, opening at a higher elevation than the road: a wide central gallery (gallery B) and two small lateral galleries (galleries A and C).

Fumane stratigraphic sequence includes about 12 meters of deposit with occupations by Homo neanderthalensis and Homo sapiens. This sequence was initially divided into four macro-units, distinguished based on geological substrate, lithological characteristics, and the type and organization of the archaeological content (Fig. SI1.1)(Cremaschi and Ferraro, 2006). From the bottom, above the residual dolomitic sands, there is:

1. Macro-unit S, formed by sands derived from the disintegration of dolomitic limestone, loess, and cryoclastic materials with smoothed edges, mostly autochthonous limestones. The macro-unit is articulated into levels initially inclined outward from the cavity and later planar. There are Mousterian occupation levels with combustion structures, possibly dated to the Middle Pleistocene. The upper boundary of the macro-unit is clear.
2. Macro-unit BR, characterized by the presence of thermoclastic breccia mixed with loess, and the absence of residual sands. There is no evidence of colluvial inputs while erosion activities could be the origin of a discontinuity between BR7 and BR6 units. The units are organized into a slightly inclined planar stratification outward from the cavity and show minor anthropization except for the inhabited soil of the oldest unit, BR 11. The upper boundary is clear and planar.
3. Macro-unit A, distinguished from the previous one mainly based on intense Mousterian (units A12-A4), Uluzzian (A3), and Aurignacian (A2-A1) occupations. It is composed of horizontal levels of thermoclastic breccia with fine fractions represented by residual sands from dolomitic limestone dissolution in the lower part (A13-A12) and eolian silts in the upper part (A11-A1). The different incidence of these main granulometric fractions and the variation in organic matter content allow the subdivision of the unit into numerous anthropic occupation levels; these appear as dark strata (centimeter-decimeter thick) interspersed with lighter strata with very scarce anthropic inputs. The upper boundary is flat and clear.
4. Macro-unit D, closes the sequence and consists of debris that blocked access to the cave and filled the main cavity and the two secondary galleries. This unit, characterized by the presence of differentiated sedimentary bodies or large boulders accumulated and slipped inward and outward from the cave, is attributed to three main detrital episodes caused by macrogelivation alternated with episodes of eolian and colluvial sedimentation and pedogenesis. These episodes would have occurred between MIS 3 and MIS 2 and perhaps later; from level D3a, there is a significantly wetter phase that favors the formation of an incipient soil: here Aurignacian anthropization is still evident, while later (probably during Gravettian) it becomes sporadic.

The main late Mousterian unit concerned in this work is A9. This is a unit formed by thermoclastic breccia with low loess content, rich in organic matter. Investigated throughout the atrial area of the cave for at least 60 square meters, it presents itself as an alternation of flat anthropized levels (A9, A9I, A9II, A9 base) characterized by lithic artifacts, bone fragments, and numerous charcoals, and with fine sandy lenses from 4 to 0.5 cm thick, with weaker anthropization. The anthropized levels have numerous charcoal accumulations likely due to the presence of structures or combustion-derived features, totaling about fifty. A8, present exclusively towards the exterior, at the cave entrance, is considered a facies of unit A9. The upper boundary with A7, a generally sterile unit, is clear, sub-horizontal, and regular.


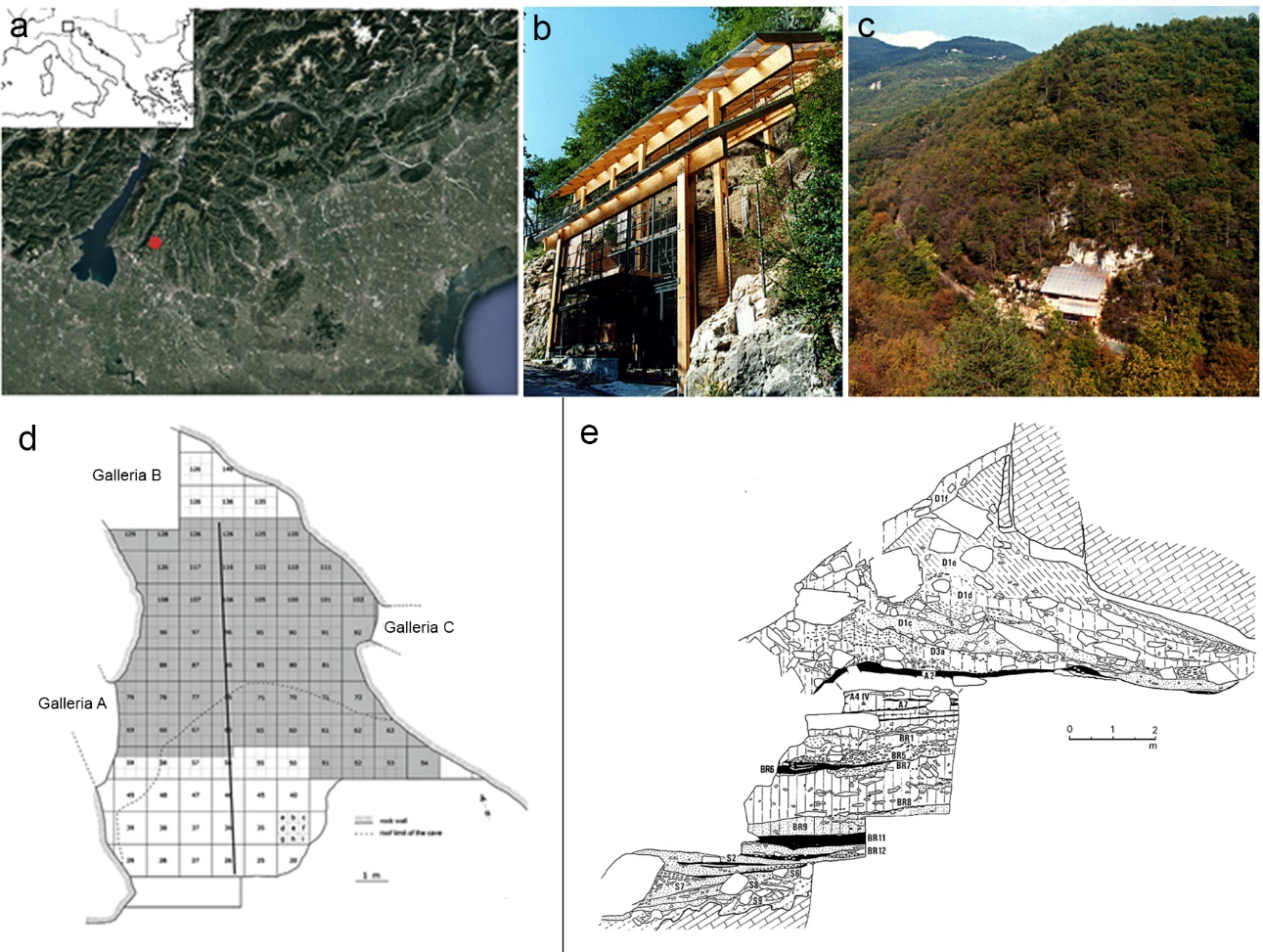


Figure SI1.1. Localization of Fumane Cave in north-eastern Italy (a); view of the site (b) within the valley (c); planimetry of the cave with positioning of the stratigraphic sequence and excavated area of A9 unit (d); stratigraphic sequence in longitudinal section (e).

1.2 Mousterian lithic industry

Within the Mousterian sequence, periods of substantial technological continuity are recorded, as well as moments where technological and cultural alternations and shifts are sometimes abrupt and clear (Peresani, 2012). The entire lower portion of the stratigraphy, meaning the levels corresponding to macro-unit S and the lower part of macro-unit BR (from S9 to BR7), is characterized by the dominance of the Levallois knapping method in its recurrent centripetal and unidirectional or preferential variants. This method is mostly exclusive or, in any case, the most frequent system for producing flake blanks. However, in one of these levels (BR9), three bifacial tools obtained from the working of blocks, which were introduced to the site already finished, were found. The lower levels of macro-unit BR are also characterized by minor alternative productions such as superficial exploitation of cores reminiscent of the Discoid and Quina methods, as well as the Kombewa exploitation of flake cores.

The first clear change with the replacement of the primary knapping method occurs between levels BR6 and BR4, where the Quina volumetric system was recognized for the fabrication of flake blanks. In these levels, the rate of flake transformation into retouched tools is decidedly high, making it difficult to recognize the knapping methods. In any case, technical characteristics on the blanks and the typological framing of the assemblages, rich in Quina and demi-Quina scrapers, indicate a shift from the Levallois system to the Quina system.

Levels that are sparsely anthropized follow BR4, up to the appearance of macro-unit A and particularly the very rich level A11, where the Levallois knapping method becomes predominant again, with the coexistence of recurrent centripetal and unidirectional variants.

*Lithic assemblage of A9 unit*

Excavated over a surface of more than 68 square meters, Unit A9 has yielded a very rich lithic industry with almost 9,000 pieces, considering artifacts whose sum of the two major axes is at least 4 cm. Unit A9 records the definitive appearance of the Discoid knapping method, used exclusively, and is particularly evident given its position between two predominantly Levallois units, A10 below and A6 above. Studies on the lithic assemblage of Unit A9 have highlighted the technological variability of the Discoid Mousterian, revealing that the technological system was structured into two reduction sequences: the most common involved the reduction of blocks, while the second, with a less productive approach, aimed at the exploitation of flake cores. The common goal of both sequences was to produce short, robust, and sometimes pointed artifacts, such as pseudo-Levallois points, débordant flakes, and sub-circular, squared, or triangular centripetal flakes (Peresani, 1998).

The main reduction sequence exploited blocks, slabs, and nodules, while the secondary one used flakes originating from either by-products of knapping (cortical flakes) or introduced directly into the site after targeted procurement or reduction strategies (Delpiano et al., 2018). The cores began to provide usable blanks from the initial stages, with flakes gradually modifying their profile and knapping pattern from unidirectional to Discoid, in a continuous and centripetal cycle aimed at maintaining and accentuating the convexities of the cores for productive purposes (Fig. SI1.2). The flakes became progressively shorter, and simple patterns with one or two parallel or converging removals gave way to more developed centripetal patterns. Additionally, modifications in the functional and morphological arrangements and convexities of the core surfaces were recorded, with their shapes and volumes tending towards polyhedral in the end.


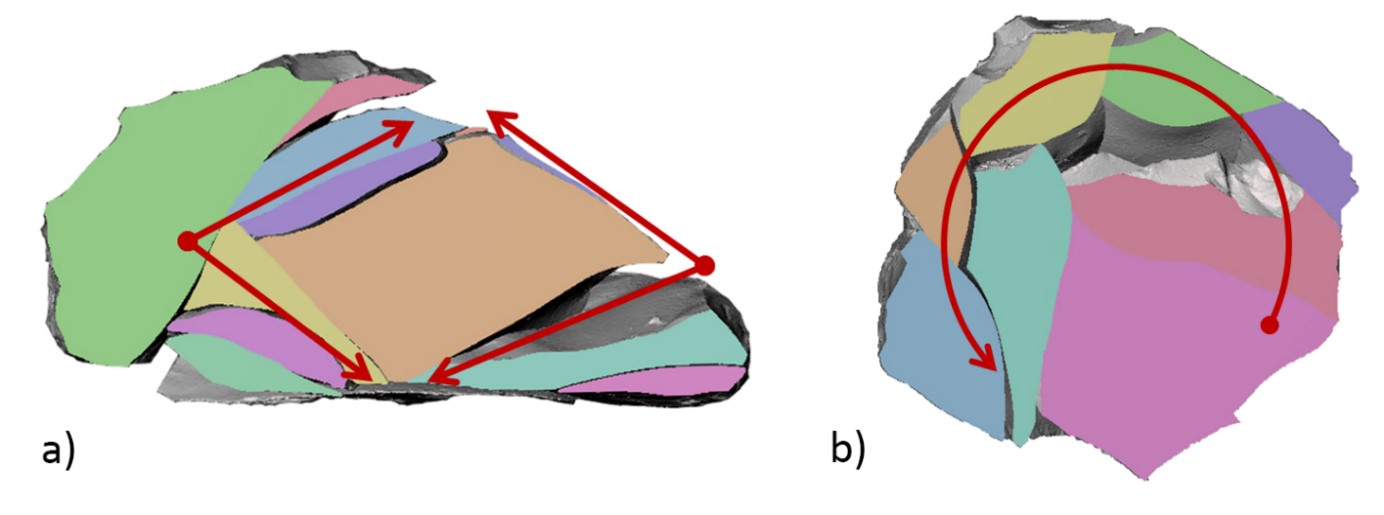


Figure SI1.2. Discoid Technology of Fumane shown through the sections of a 3D refit. The alternate exploitation of the two core surfaces (a) and the continuous centripetal reduction pattern (b) are evident. Modified from Delpiano and Peresani, 2017.

This type of planning in the organization of operational chains would lead to obtaining a wide range of blanks that meet a variety of needs. Data obtained from a functional study on samples from Unit A9, however, show that, although with some exceptions, different types of activities and related movements/actions are randomly distributed throughout the sample. Moreover, the different types of blanks, both retouched and unretouched, were used similarly in the processing of materials from hard to moderately hard/soft rather than from hard to very hard. The movements involved in these activities are variable, with transverse movement being more frequent than longitudinal, unidirectional, and bidirectional movements. Given the considerable thickness of the analyzed blanks (mean value = 11 mm), the interpretation of Discoid artifacts, although distorted by a partial alteration of the potentially workable edges, is that of robust and multifunctional blanks, suitable for the processing of moderately hard materials (Lemorini et al., 2003).

The lithic artifacts were produced using flints from different sources, with the most closely local resources greatly influencing the lithic assemblage. However, targeted use of semi-local raw materials (mainly oolitic and Eocene flints) that outcrop in primary or secondary deposits in the area between 5 and 10 km in a straight line from the site has been recognized; these materials were introduced already partially worked as cores in a mid-advanced stage of reduction, or as mobile flake blanks, and reduced according to a Discoid technology applied to flake cores (Kombewa type) or in more unidirectional ways (Delpiano et al., 2018).

The rare, though present, use of allochthonous raw materials (Rosso ad Aptici formation), combined with the presence of a fossil gastropod, both collected from sources located at least 80 km and 110 km away respectively from the site, along with evidence of recycling old patinated artifacts, indicate a complex and diversified use of raw materials, linked to varied mobility strategies and including opportunistic reuse of materials abandoned in previous occupations and the logistical planning of techno-economic organization based on the quality and distance of available raw material sources (Delpiano et al., 2018; Peresani et al., 2015, 2013).

1.3 Archaeozoological data

The faunal assemblage of Unit A9 includes over 100,000 remains, of which 1,259 large and medium-sized mammals have been identified at least to the family level (Romandini et al., 2014). The number of identified remains is low due to high fragmentation (over 92.5% of fragments are smaller than 2 cm) and combustion rates (50%). These conditions are due to a series of depositional and post-depositional processes also observed in other fully anthropic contexts. However, carnivores are also common agents of bone destruction and accumulation in Paleolithic assemblages. In A9, the frequency and patterns of gnawing traces by rodents and carnivores suggest secondary access to ungulate remains, indicating that carnivores did not significantly contribute to the bone accumulation. Regarding large carnivores, those identified in the assemblage (lion, hyena, cave bear) did not leave macroscopic traces on the bones.

The hunting activity was not specialized in one or more selected taxa but rather adapted to the availability of prey in the western Lessini. Well-established and efficient patterns were applied in the meat exploitation of carcasses. Deer and roe deer, the two most common species with over half of the identified remains, indicate wooded environments interspersed with open spaces. The presence of moose, aurochs, and wild boar suggests that wet areas with water sources existed in a generally temperate cold climate context. Bisons and giant deers would have needed access to open and sparsely wooded environments, which could be found in the plain or above the forest belt (between 700 and 1000 m a.s.l.) not far from the site. This latter environment was also vital for chamois and ibex, well-documented in the assemblage. The high incidence of cervids is consistent with the general faunal record of the Mousterian stratigraphic sequence (Cassoli and Tagliacozzo, 1991; Fiore et al., 2004), which shows a progressive increase of these species from units A11-A10 at the expense of those preferring open environments.

The complex of Unit A9, similar to other contexts along the same stratigraphic sequence (Peresani et al., 2011), records the presence of hundreds of avifaunal remains, some still in anatomical connection. The age ranges determined by dental wear analysis and the state of epiphyseal fusion reveal a predominance of adults and mature adults, while relatively few are pre-adult individuals. The scarcity of fetal and neonatal individuals makes it impossible to identify a preferential seasonal use of the site. A fragment of a young bison mandible with skinning marks is the only indication that the capture occurred in spring (Romandini et al., 2014).

Anthropic traces produced during the carcass butchering are observed on all types of ungulates. In particular, large cervids and large bovids show traces from the few cranial elements present to the more abundant limbs (especially the hind limbs). Cuts made with lithic tools are the most common trace and are often repeated, especially on long bones and at points corresponding to muscle and tendon insertions. Striations on cranial or appendicular elements suggest skinning. Longitudinal scrapes on the diaphyses suggest that the periosteum was removed to clean the bone from meat residues and prepare it for intentional breakage. Single or multiple percussion marks, percussion pits, and spiral fractures typical of fresh bone fracture were also observed. The impact points are mainly on long, thick bones resistant to trauma, such as tibias and distal humeri, metapodials, and in some cases, phalanges. Numerous diaphysis fragments were used as hammers to retouch flint artifacts (Jéquier et al., 2013; Martellotta et al., 2020).

Ungulates, divided by their respective anatomical elements, show a clear prevalence of limb fragments (over 60%), followed by extremity elements such as phalanges and sesamoids (14.7%). Cranial remains are scarce and predominantly represented by isolated teeth (11.5%) and parts of the hemimandibles and maxilla (4.4%). The trunk and vertebral column are almost absent (1.8%). This bias becomes evident for large cervids (moose and giant deer), and even more pronounced in the case of bisons and aurochs, but is also noted in smaller ibex and chamois. Therefore, the site was used as a habitation place where carcass processing was completed after being initiated at the kill site. This is clearly indicated by the preservation pattern of the skeletal elements, from which it is possible to deduce that groups of humans transported selected anatomical portions of high nutritional value (both marrow and meat), such as limbs, especially the hind limbs, and to a lesser extent, the skull, to the site, in an economical view of the cost-benefit ratio related to transportation (Romandini et al., 2014).

1.4 Environmental data and chronology

Various sources of data have contributed to the reconstruction of the ecological framework of the late Mousterian sequence at Fumane Cave. Data on micro-mammals and large mammals indicate temperate and relatively humid conditions accompanied by an expansion of tree cover and woodland habitats from A11 to A9, as evidenced by the abundance of ungulate species, European deer, and roe deer (Fiore et al., 2004; López-García et al., 2015; Romandini et al., 2014). The ecological structure shown by the avifaunal assemblage reflects various environments, including rocky cliffs and alpine meadows, mountainous areas and ponds, high mountain woods, and subalpine forests (Fiore et al., 2016). Additionally, an anthracological investigation conducted on the charcoals from A9 confirms the presence of larch, spruce, pine, and birch (Basile et al., 2014). In particular, the sequence from A11 to A9 shows a peak of wet environments in A10 and a peak in temperatures in A9.

Considering the 14C dates obtained from the overlying units, the known inaccuracies due to sample contamination in this age range, and the ESR result, it is likely that the oldest of the six 14C dates (47,600 calibrated years BP) is the most reliable minimum age for Unit A9 (Peresani et al., 2008). A sampling campaign to obtain new datings from different methods is currently underway and includes both the lower A units (A9 – A10 – A11) and the underlying BR units.

Unit A9 also preserved a fragmentary marine shell from Miocene-Pliocene formations, Aspa marginata. According to paleontological and taphonomic data, it has been confirmed that the shell was collected by Neanderthals from a fossil outcrop probably located more than 110 km from the site, from Miocene and Pliocene outcrops along the foothills south of the Po Valley. The nearest fossil shells of Aspa marginata are also reported as occasional finds in Veneto, near Cornuda and Anzano di Vittorio Veneto, and in the Lombardy region. Traces of ochre, striations on the inner lip, and other aspects suggest that the shell was modified to be used as a pendant (Peresani et al., 2013).

Finally, the human remains (two deciduous incisors from A9 and a deciduous molar from A11) demonstrate the presence of at least one Neanderthal infant in A9 and a young individual in A11, suggesting the presence of the entire human group (Benazzi et al., 2014).

1. **La Rochette**

2.1 Research and stratigraphy

The La Rochette rockshelter is located in the municipality of Saint-Léon-sur-Vézère, in Dordogne (Fig. SI1.3). Research, initially carried out rather summarily, began in the first half of the 20th century with excavations directed first by Otto Hauser and then by M. Coutier. However, the site was partially removed by an adjacent stone quarry. In the 1960s, Delporte described it as an “archaeological ruin,” an entanglement of trenches, holes, tunnels, and fragments of archaeological layers, also resulting from frequent unauthorized excavations. It is an extensive and rich site, mainly focused on the central shelter (sector B by Delporte, 1962).


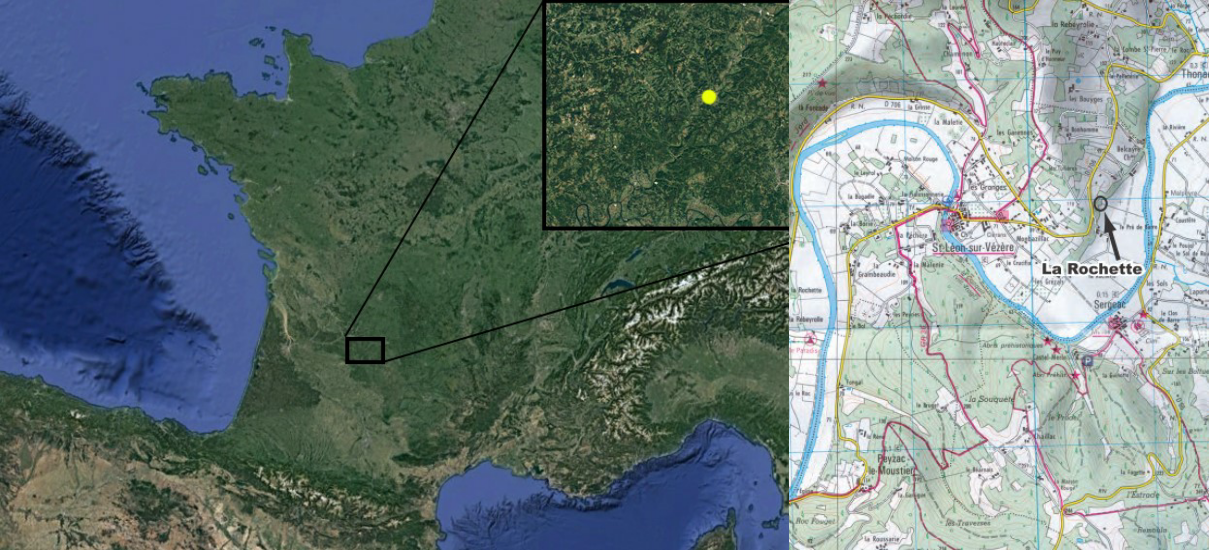


Figure SI1.3. Localization of La Rochette rockshelter in south-western France within Vézere valley.

The stratigraphic sequence, about 3.5 meters thick in the central sector, also includes recent deposits, the partial collapse of the shelter’s vault, and the “tunnel” subject to Otto Hauser’s excavations (Fig. SI1.4). Delporte's research defined a complex stratigraphy comprising levels dating to the Upper and Middle Paleolithic. Specifically, below level 1 (modern topsoil) and level 2 (collapsed boulders), levels 3, 4, and 5 belong to a presumed evolved Aurignacian (Aurignacian II), with carinated scrapers-cores, abundant burins without typical beaks, and numerous Dufour bladelets, many of which have alternate retouch (Delporte, 1963). To the south, L. Coutier found an Aurignacian I, with Aurignacian blades, strangled blades, split-base and lozenge bone points, but this occupation level, 20 cm thick, rapidly decreased toward the east. Also to the south, the Aurignacian was covered by a Gravettian level with Noailles burins (Upper Perigordian according to Peyrony), with possibly truncated elements, while D. de Sonneville Bordes reported the presence of Solutrean tools (Soressi, 2002).


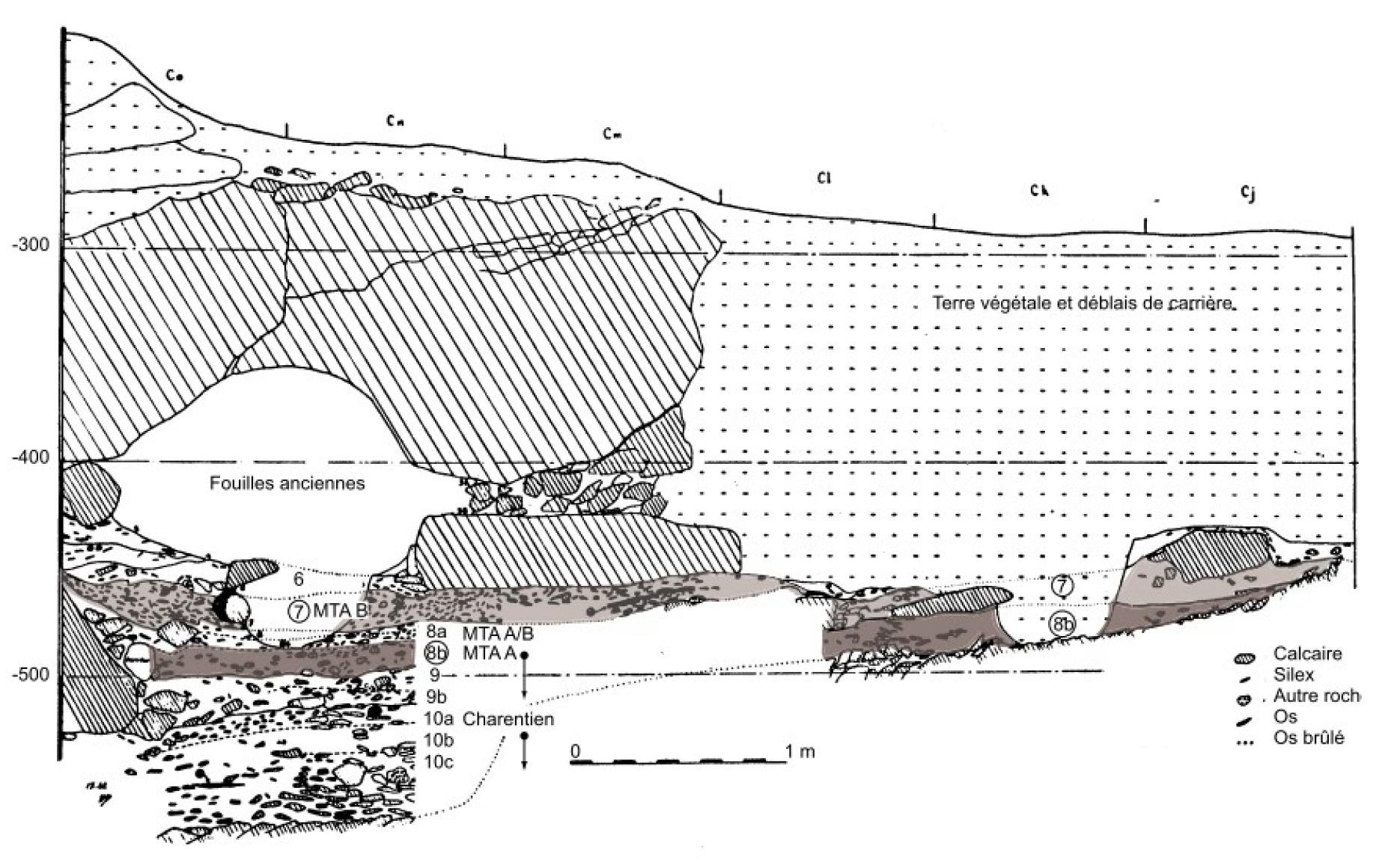


Figure SI1.4. Stratigraphical sequence of La Rochette rockshelter (Modified from Soressi, 2002).

Below, the description of the levels related to the transition to the Middle Paleolithic and the Mousterian:

- Level 6: a consolidated yellowish layer in breccias with small black and red nodules. Presence of rounded limestone elements, as well as flint that is shiny and shows traces of rolling and crushing. This evidence suggests that a post-depositional disturbance of this level, such as rill wash, is the origin of its formation. The lithic industry indicates a close relationship with the underlying level 7, from which the pieces probably originate (Delporte and David, 1966).
- Level 7: a black layer, 15 to 20 cm thick, containing some limestone globular elements. The lithics are generally in good condition but locally there are smoothed pieces with a light patina; thermally altered flints are common, sometimes fractured by heat. The industry has been placed within the Mousterian of Acheulean Tradition type B (MTA-B) (Delporte and David, 1966).
- Level 8: a dark clayey layer corresponding to the top of a 15-20 cm thick unit and divided into further levels based on variations in sediment color and structure. The fauna is almost absent and the lithics, shiny and smoothed, are fewer than in the overlying level 7, but they still follow the characteristics of MTA-B.
- Level 8a: a lighter layer with a sandier matrix with rounded breccia skeleton and few flints showing clear traces of post-depositional movements; the material might come from the top of the underlying layer and the base of level 8. For this reason, the industry would resemble level 5 of Pech de l'Azé I, characteristic of a transition between MTA-A and MTA-B. It might correspond to the sterile layer in O. Hauser’s stratigraphy.
- Level 8b: a brown level with an undisturbed zone characterized by the probable presence of a hearth. The lithic industry recalls the Mousterian of Acheulean Tradition type A.
- Level 9: a yellow layer with a clay-sandy matrix with small rounded gravel. It is 16-22 cm thick and has some gaps. The industry, homogeneous and fresh, follows the MTA-A tradition, though it is relatively poor.
- Level 10: a brown-reddish sandy layer with clear boundaries with the overlying level, also marked by the presence of a level with limestone slabs. It becomes significantly thicker (55 cm) towards NW, where it was divided into three sub-units: 10a, 10b, and 10c. The lithic industry is notably fresh and records a Charentian type Mousterian, in stark contrast with the upper series.

2.2 Environmental data and chronology

The first 14C datings of level 7 date back to the 1960s, but unfortunately, they were obtained from burned bone. These dates would place the occupation at 36,000 years ± 550 (GrN 4362) and 30,700 years ± 400 (GrN 4345) (Vogel and Waterbolk, 1967). Subsequently, additional dates were obtained on samples of unburned bones (large mammal diaphysis fragments) from level 7, in an area (square Cm 7) where level 7 is sealed by large limestone slabs and therefore more reliable, also due to the richness of the material. The dates are at the extreme limits of the C14 method, with one even reaching 52,500 ± 3400 calibrated years BP, which should be the date closest to the Neanderthal occupation. Level 7 should be at least 50,000 years old, even though it represents the last documented level of Neanderthal occupation on site, given the scarcity of material from level 6, which appears to be connected to level 7.

From a paleoenvironmental perspective, data from fauna and botanical remains, as well as geomorphological analyses, are practically absent at the moment. In level 7, the presence of fauna is poorly documented: there are reindeer remains (though still a minority) and a greater number of bovids. Reindeer, in fact, become dominant in the Aurignacian. In level 10, reindeer presence is also poorly documented, while there are horses and bovids. The nature of the sediment tends to suggest formation in a fairly humid environment (Delporte, 1962).

The “MTA” occupation, in accordance with the available chronology, should have occurred at a relatively early time in isotopic stage 3, possibly around the Heinrich 6 (H6 – about 54,000 years ago) and H5 – about 45,000 years ago. During this phase, there should not have been any abrupt paleoenvironmental changes in the Dordogne area, which, as suggested by the high density of contemporary archaeological sites, should have provided a great quantity of natural resources, ranging from lithic to food resources in the form of large ungulates.

- 1. Mousterian lithic assemblages and layer 7

Hauser distinguished two lower levels, one with bifacial tools and one without. The first was classified as Acheulean, partly because at the time the MTA had not yet been defined. The second was classified as Mousterian, later corrected to typical Mousterian by Peyrony himself. In her doctoral thesis, Soressi (2002) suggested that Hauser's Acheulean level most likely corresponds to Delporte's levels 8b and 9, and that the Mousterian level identified by Hauser corresponds to Delporte's levels 7 and 8, bolstered by the proximity of the two excavated sectors.

In the MTA-A (levels 8b and 9), bifacial tools are particularly present, with over 200 specimens in O. Hauser's collection alone. These were also made on large flakes, which in turn allowed for the production of usable flakes that also helped shape the tool (more than 50 per bifacial tool). The longer ones were indeed retouched into scrapers, specialized tools for low-intensity activities, while the bifacial tools served as mobile and multifunctional supports. Already in these levels, the flake production methods included the semi-turning unipolar method and a sort of "simplified" Discoid method with secant face exploitation.

In MTA-B and especially in level 7, which evidences the richest occupation, Levallois débitage is sparsely represented; the butts are generally plain even among retouched tools, with few faceted ones. The knapping angles are quite open, while the flake blanks are thick with a laminar tendency. Levallois cores, like the products of knapping, are almost absent, whereas Discoid and globular or irregular cores are present. Among the tools, scrapers are rare, bifacial tools are almost absent, while denticulated tools, pseudo-Levallois points, and especially backed knives are common; these are both typical, atypical, and natural, produced on flakes and blades through deep or marginal retouch. There is also a minor presence of Upper Paleolithic tools, such as atypical burins and some truncations.

Soressi's (2002) analysis better defined the most recurrent débitage system in the MTA, particularly in level 7 at La Rochette: the unipolar volumetric method. This semi-turning concept starts from the exploitation of a dihedral on a narrow front and then expands on the adjacent surfaces. There are débordant flakes detached transversely, allowing for the removal of parts of the crests and distal convexities and starting again with other transverse unipolar series, forming polyhedral-globular cores. Sometimes production ends with orthogonal or centripetal débitage. Knapping is always carried out with direct percussion using a hard hammer, and the flakes used (mainly long, laminar, triangular-sectioned, half symmetrical and half asymmetrical flakes) are sometimes transformed into retouched tools: approximately one-third are notches, one-third denticulated (made on wider and shorter flakes), and one-third backed knives (made on symmetrical unipolar blades or flakes). Used flakes (defined based on macro-traces) were also taken from short, wide, asymmetrical, and/or orthogonal/centripetal products. Soressi's (2002) conclusion is that a wide set of blanks, even very different from each other, were used in a targeted and differentiated manner. To use the elongated, sufficiently wide, and symmetrically triangular-sectioned products, it is necessary to knock off the back, sometimes only marginally. There are natural backed knives obtained from the initial knapping stages of the blocks, where the unipolar system is already evident, as well as backed knives derived from managing convexities and unipolar or orthogonal-centripetal production on cores. In these cases, the cortex and the thick knapping surface allow for gripping, which is not possible for unipolar blanks characterized by two raw cutting lateral margins. Retouch is therefore used to increase the number of manageable pieces.

Regarding territorial mobility, the lithic industry does not provide particular insights as it was produced exclusively from strictly local raw materials, mainly Senonian flint (in some cases gray flint and quartz) sourced from Vézère alluvium. In fact, traces of river cortex have been recognized in several cases (42% of pieces in Soressi, 2002). There is also a high presence of cortical or semi-cortical pieces, including *entames*, indicating that all knapping phases were carried out on site, and that raw or tested/lightly trimmed blocks were introduced. This shows a difference from the levels with MTA-A industry, where knapping activities were organized territorially, mainly due to the introduction and abandonment of highly mobile blanks with high potential use like bifacial tools. In that case, long-term planning of activities was reflected in the territorial fragmentation of knapping, while in MTA-B this organization is short-term and more local and immediate.

**Bibliography**

Basile, D., Castelletti, L., Peresani, M., 2014. Results from the anthracological investigation of the Mousterian layer A9 of. Quartar 61, 103–111. https://doi.org/10.7485/QU61

Benazzi, S., Bailey, S.E., Peresani, M., Mannino, M.A., Romandini, M., Richards, M.P., Hublin, J., 2014. Middle Paleolithic and Uluzzian human remains from Fumane. J. Hum. Evol. 70, 61–68. https://doi.org/10.1016/j.jhevol.2014.03.001

Cassoli, P.F., Tagliacozzo, A., 1991. Considerazioni paleontologiche, paleoecologiche e archeozoologiche sui macromammiferi e gli uccelli dei livelli del Pleistocene superiore del Riparo di Fumane (VR) scavi 1988/1991. Boll. Mus. Civ. Stor. Nat. Verona 23, 85–117.

Cremaschi, M., Ferraro, F., 2006. The Fumane rockshelter - palaeoclimatic signiﬁcance of the stratigraphic sequence, in: Donegana, M., Ravazzi, C. (Eds.), Quaternary Stratigraphy and Evolution of the Alpine Region in the European and Global Framework. The Quaternary of the Italian Alps. Field Trip Guide. INQUA-SEQS, pp. 137–142.

Delpiano, D., Heasley, K., Peresani, M., 2018. Assessing neanderthal land use and lithic raw material management in discoid technology. J. Anthropol. Sci. 96, 89–110. https://doi.org/10.4436/jass.96006

Delpiano, D., Peresani, M., 2017. Exploring Neanderthal skills and lithic economy. The implication of a refitted Discoid reduction sequence reconstructed using 3D virtual analysis. Comptes Rendus - Palevol 16. https://doi.org/10.1016/j.crpv.2017.06.008

Delporte, H., 1963. Le passage du Moustérien au Paléolithique supérieur., in: Aurignac et l’Aurignacien. Centenaire Des Fouilles d’Edouard Lartet. Bulletin de la Société Méridionale de Spéléologie et Préhistorique, pp. 40–50.

Delporte, H., 1962. Le gisement paléolithique de La Rochette (commune de Saint-Léon-sur-Vézère, Dordogne). Gall. préhistoire 5, 1–22. https://doi.org/10.3406/galip.1962.1202

Delporte, H., David, R., 1966. L’évolution des industries moustériennes à la Rochette, Commune de Saint-Léon-sur-Vézère (Dordogne). Bull. la Société Préhistorique Française 42, 48–62.

Fiore, I., Gala, M., Romandini, M., Cocca, E., Tagliacozzo, A., 2016. From feathers to food : Reconstructing the complete exploitation of avifaunal resources by Neanderthals at Fumane cave , unit A9 From feathers to food : Reconstructing the complete exploitation of avifaunal resources by Neanderthals at Fumane cave , unit . Quat. Int. https://doi.org/10.1016/j.quaint.2015.11.142

Fiore, I., Gala, M., Tagliacozzo, A., 2004. Ecology and subsistence strategies in the Eastern Italian Alps during the Middle Palaeolithic. Int. J. Osteoarchaeol. 14, 273–286. https://doi.org/10.1002/oa.761

Jéquier, C., Nannini, N., Romandini, M., Peresani, M., 2013. Assessing the role of bone retouchers from the Mousterian to the Aurignacian in the North of Italy., in: PROCEEDINGS OF THE EUROPEAN SOCIETY FOR THE STUDY OF HUMAN EVOLUTION.

Lemorini, C., Peresani, M., Rossetti, P., Malerba, G., Giacobini, G., 2003. Technomorphological and use-wear functional analysis: an integrated approach to the study of a discoid industry, in: Peresani, M. (Ed.), Discoid Lithic Technology. Advances and Implications. British Archaeological Reports International Series, pp. 257–275.

López-García, J.M., dalla Valle, C., Cremaschi, M., Peresani, M., 2015. Reconstruction of the Neanderthal and Modern Human landscape and climate from the Fumane cave sequence (Verona, Italy) using small-mammal assemblages. Quat. Sci. Rev. 128, 1–13. https://doi.org/10.1016/j.quascirev.2015.09.013

Martellotta, E.F., Delpiano, D., Govoni, M., Nannini, N., Duches, R., Peresani, M., 2020. The use of bone retouchers in a Mousterian context of Discoid lithic technology. Archaeol. Anthropol. Sci. 12, 228. https://doi.org/10.1007/s12520-020-01155-6

Peresani, M., 2012. Fifty thousand years of flint knapping and tool shaping across the Mousterian and Uluzzian sequence of Fumane cave. Quat. Int. 247, 125–150. https://doi.org/10.1016/j.quaint.2011.02.006

Peresani, M., 1998. La variabilité du débitage discoïde dans la grotte de Fumane (Italie du Nord). Paléo 10, 123–146. https://doi.org/10.3406/pal.1998.1133

Peresani, M., Boldrin, M., Pasetti, P., 2015. Assessing the exploitation of double patinated artifacts from the Late Mousterian : Implications for lithic economy and human mobility in northern Italy. Quat. Int. 361, 238–250. https://doi.org/10.1016/j.quaint.2014.10.058

Peresani, M., Cremaschi, M., Ferraro, F., Falguères, C., Bahain, J.J., Gruppioni, G., Sibilia, E., Quarta, G., Calcagnile, L., Dolo, J.M., 2008. Age of the final Middle Palaeolithic and Uluzzian levels at Fumane Cave, Northern Italy, using 14C, ESR, 234U/230Th and thermoluminescence methods. J. Archaeol. Sci. 35, 2986–2996. https://doi.org/10.1016/j.jas.2008.06.013

Peresani, M., Fiore, I., Gala, M., Romandini, M., Tagliacozzo, A., 2011. Late Neandertals and the intentional removal of feathers as evidenced from bird bone taphonomy at Fumane Cave 44 ky B.P., Italy. Proc. Natl. Acad. Sci. U. S. A. 108, 3888–3893. https://doi.org/10.1073/pnas.1016212108

Peresani, M., Vanhaeren, M., Quaggiotto, E., Queffelec, A., D’Errico, F., 2013. An Ochered Fossil Marine Shell From the Mousterian of Fumane Cave, Italy. PLoS One 8, e68572. https://doi.org/10.1371/journal.pone.0068572

Romandini, M., Nannini, N., Tagliacozzo, A., Peresani, M., 2014. The ungulate assemblage from layer A9 at Grotta di Fumane, Italy: A zooarchaeological contribution to the reconstruction of Neanderthal ecology. Quat. Int. 337, 11–27. https://doi.org/10.1016/j.quaint.2014.03.027

Soressi, M., 2002. Le Moustérien de tradition acheuléenne du sud-ouest de la France. Doctoral Thesis, Université de Bordeaux.

Vogel, J., Waterbolk, H., 1967. Groningen Radiocarbon Dates VII. Radiocarbon 9, 107–155.
